# Exploring drivers of overnight stays and same-day visits in the tourism sector

**DOI:** 10.1038/s41598-024-60229-w

**Published:** 2024-04-29

**Authors:** Francesco Scotti, Andrea Flori, Piercesare Secchi, Marika Arena, Giovanni Azzone

**Affiliations:** 1 Department of Management, Economics and Industrial Engineering, Politecnico di Milano, Via Lambruschini, 4/B, 20156 Milan, Italy; 2 Impact, Department of Management, Economics and Industrial Engineering, Politecnico di Milano, Milan, Italy; 3 MOX - Dipartimento di Matematica, Politecnico di Milano, Piazza Leonardo da Vinci 32, 20133 Milan, Italy

**Keywords:** Mobile data, Tourism, Visitors, Gravity model, Network analysis, Socioeconomic scenarios, Scientific data, Statistics

## Abstract

We employ mobile network data referred to the area of Lombardy in Italy to investigate alternative touristic behaviours, such as same-day visits and overnight stays in Italy. We show that larger availability of tourism accommodations, cultural and natural endowments are relevant factors explaining overnight stays. Conversely, temporary entertainment and transportation facilities increase municipalities attractiveness for same-day visits. The results also highlight a trade-off in the capability of municipalities of being attractive in connection to both the tourism behaviours. For instance, higher tourists arrivals are observed in areas receiving limited visitors, coming from municipalities with low same-day visits outflows. We highlight mobile data offer an adequate level of spatial and temporal granularity and can be thus employed to support policy makers in the design of effective tourist management strategies.

## Introduction

Tourism length of stay constitutes a pivotal concern in tourism demand management since it significantly affects the economic, social and environmental impact of tourism related activities^1–6^. Consistently, the literature on tourism management has formalized the concepts of overnight *tourists* and same-day *visitors* as two alternative tourism behaviours^7^. More specifically, tourists are delineated as individuals who venture to locations distinct from their usual residence, engaging in overnight stays^7,8^. Conversely, visitors are defined as people experiencing a same-day visit at a destination^9,10^.

The analysis of dynamics behind tourists and visitors behaviours may support policy makers to design more effective socio-economic development strategies. First, tourists and visitors may have a different economic impact at local level, since the former allow accommodation infrastructures to obtain higher occupation rates, reduce fixed costs, and achieve larger returns^11–14^. Second, the two groups may make a different usage of available infrastructures, with overnight tourists requiring accommodations such as hotels, resorts, or guesthouses, while one-day visitors are more interested in adequate parking facilities and transportation options^15,16^.

Also in terms of destination management, distinct strategies could be put in place. Indeed, overnight tourists may ask for access to a wide set of attractions, amenities and cultural events which necessitate a specific effort in terms of tourism planning and management activities. Conversely, one-day visitors may require well-designed visitor centers and properly managed day-use facilities. Finally, overnight tourists and one-day visitors can have diverse environmental footprints since the different stay duration may imply heterogeneous waste generation, water, energy consumption and CO_2_ emissions production^17–21^.

Despite the relevance of disentangling such alternative tourism behaviours, extant literature has not deeply investigated the main factors stimulating overnight stays and same-day visits, mainly because traditional tourism data disclosed by national statistical offices do not display temporal and spatial resolution that allow to specifically identify tourists and visitors flows (see e.g. Refs.^22–26^ and^27^). As a consequence, available studies analysing the determinants of the length of stay have only considered the flows of people spending at least one night at the destination, neglecting the phenomenon of same-day visits^28–30^.

In order to fill such gap, mobile network data may represent an useful instrument. Indeed, leveraging on information related to the most frequent position of users during days and night hours, they enable to distinguish between tourists and visitors^8,31–33^. Furthermore, they provide higher quality information with respect to other types of available data. For instance, compared to the traditional data (e.g. questionnaires and itinerary blogs), mobile network data have a higher level of resolution and reliability^34^. In addition, a comparison of tourists’ recalled diaries with mobile network data showed that the questionnaire-based data varied greatly from the real GPS data, implying there exists a bias in the traditional data that may not be adequately accurate when aiming to explore tourists’ behaviors^35^. Moreover, differently from alternative data sources such as geo-tagged photos and online diaries, that are often unavoidably inconsistent with the actual tourism behavior due to regulatory issues (such as prohibiting photographing, ethics, signal shielding of position sensors, etc.), and usually more widespread among young population cohorts, mobile network data avoid the recording of deviant and redundant information and provide a more representative overview of people flows^36^.

Against this background, our paper aims to study the heterogeneity of tourists and visitors movements in Lombardy in 2022, based on mobile network data, in order to comprehend the main drivers of these two types of tourism behaviour. Furthermore, the paper investigates the presence of potential trade-offs in the capability of municipalities to attract at the same extent both types of tourism behaviour. More in detail, we aim to answer to the following research questions:

*RQ1: Which are the main factors explaining the attractiveness of municipalities in terms of tourists and visitors flows?*

*RQ2: Is there a trade-off in the capability of municipalities to attract both tourists and visitors?*

*RQ3: Are spotted patterns stable over the whole calendar year, or do we have evidence of alternative drivers over different seasons?*

Our contributions to the comprehension of the main determinants of tourism behaviour are thus manifold. First, we highlight the relevant characteristics of municipalities fostering tourists or visitors flows. In this way, we fill a relevant gap in extant literature mainly focusing on the drivers of the length of stay of tourists, but overlooking same-day visits^37–39^. In particular, we find that one additional accommodation bed contributes to a growth of tourists flows by a percentage between 0.1% and 0.3% with respect to visitors volumes. Similarly, the presence of cultural heritage items, ski routes and natural reserves raises the same figure by a portion between 0.1% and 1.1%. On the other hand, festivals and transportation facilities such as methane distributors and intermodal nodes increase visitors presences by percentages between 0.1% and 0.6% with respect to tourists flows.

Second, based on a monthly gravity model, we provide evidence that it might be difficult for municipalities to attract at the same time overnight stays and one-day visits, as not necessarily most popular areas in terms of tourists also receive large visitors flows. For instance, municipalities experiencing higher tourists inflows tend to achieve limited visitors levels, highlighting alternative drivers motivating the two types of tourism behaviour. Conversely, nodes with a strategic position in the visitors network, bridging communities of municipalities with limited connections among them (e.g. displaying a high betweenness) result particularly attractive also for overnight stays. We discuss how such municipalities may be of particular interest, since they are able to stimulate high tourists and visitors flows at the same time.

Finally, we show that mobile phone network data display an adequate level of spatial and frequency granularity, allowing to detect relevant seasonal patterns in terms of tourists and visitors flows, thus representing a valid source of information for policy makers to design more precise local development strategies^40^.

## Data

The mobile network data used in this paper have been made available to the authors by Polis, a public entity collaborating with Lombardy region. These data are provided by a main telecommunication company in an anonymised and irreversibly aggregated form, in compliance with the privacy legislation, and the provisions of the EU GDPR, according to the Privacy by Design methodology. These data refer to the calendar year 2022 and cover 163 municipalities in Lombardy as highlighted in Fig. S1 and Table S1 in the Supplementary Information (SI). The dataset provides information about the aggregate monthly flow of individuals travelling across each couple of the analysed municipalities with details on the origin and destination of the movement.

As shown by^41^ who discuss the approach employed by the telecommunication company to use mobile network data for producing tourism-related statistics, this dataset has an adequate capability to provide a representative overview of people flows in the tourism sector. Indeed, the telecommunication company has a market share around 30% in Italy in terms of Subscriber Identity Modules (SIM). Moreover, it uses calibration factors such as the market share by geographical area and age cohort, the likelihood of not having a mobile phone or owing more than one digital device to infer statistics on the entire Italian population. Finally, proprietary machine learning algorithms are used to clean the tourism component from traditional mobility data. Reliable benchmarks such as official measures on the number of people attending sports and musical events or travelling through rail and air transport are used to validate the quality of such estimates, achieving a precision around 90% with respect to official disclosed data.

These data also distinguish among two different travelling profiles. In particular, according to the telecommunication company approach, *“visitors”* are defined as those individuals who make a visit outside the municipality of usual residence for at least four without an overnight stay. On the other hand, *“tourists”* are defined as those users with a night cell referring to a municipality that differs from the phone residence. Such definitions of these two travelling behaviours are consistent with those provided by official statistical offices. In addition, applications on the cities of Rome and Rimini show the good potential of mobile network data to integrate and complement official tourism statistics since these data also capture people flows in second houses or friends’ homes that are not detected by traditional tourism data^8^. A similar dataset has been recently employed by^42^ to analyse the impact of cultural events on crowding-in and crowding-out dynamics in the city of Venice.

Overall, the dataset accounts for 5.4 million tourists presences and 161.5 million visits in Lombardy municipalities in the calendar year 2022. Interestingly, Winter is characterized by the largest tourists flows. In particular, December achieves the highest figure with 0.5 million presences. The largest visitors flows are observed in Spring and Autumn, with May and October experiencing the largest values with around 15.5 million people flows. The lowest value is rather observed during August, with 8.8 million flows, probably due to the typical holiday period favouring overstay nights rather than short visits.

We further describe the tourists and visitors networks, by showing the geographical distribution of the two different types of flows across Lombardy municipalities.

**Figure 1:**
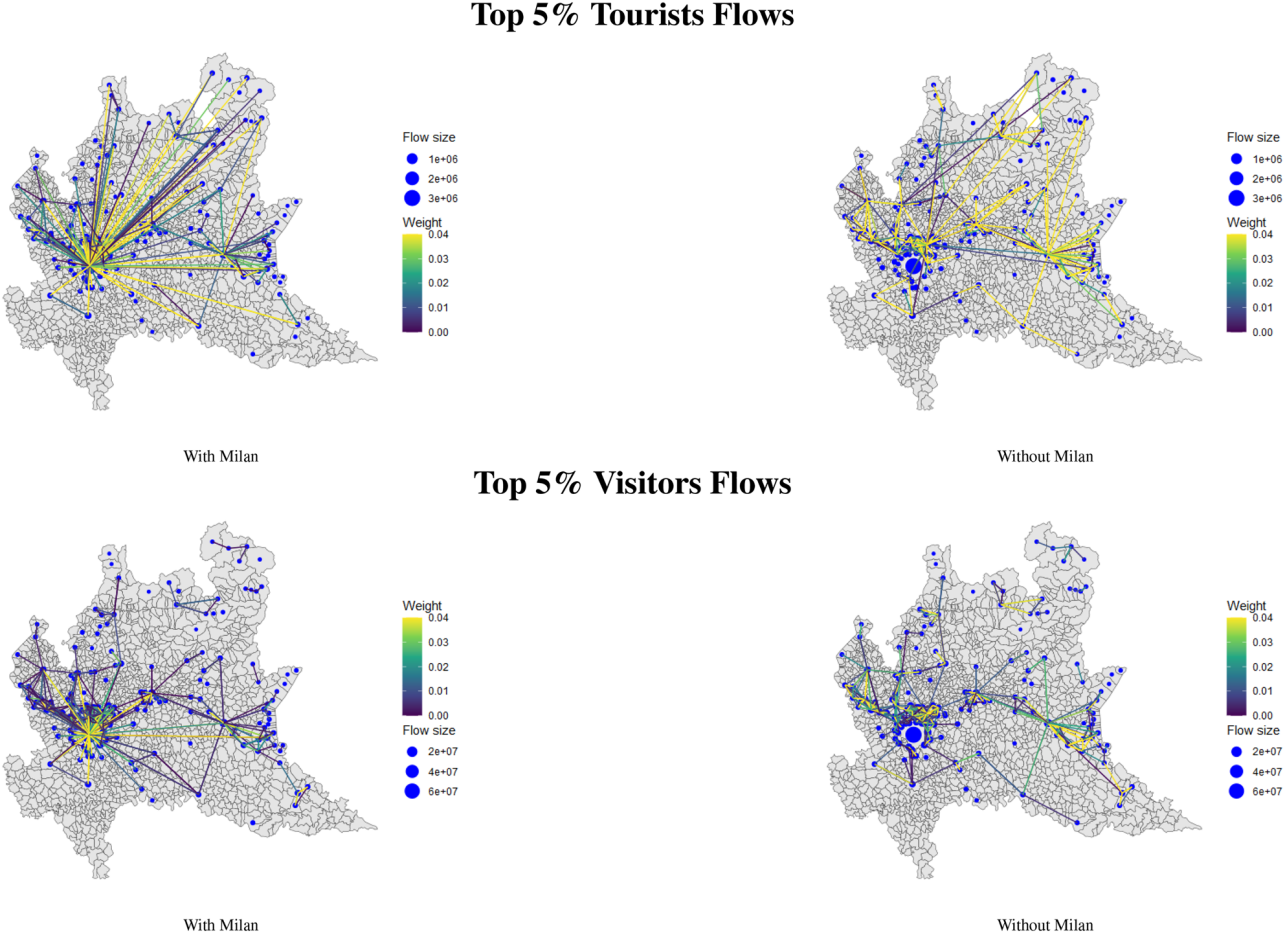
Panel top-left: we show the top 5% flows of tourists across all 163 municipalities. Panel top-right: we show the top 5% flows of tourists across municipalities excluding Milan. Panel bottom-left: we show the top 5% flows of visitors across all 163 municipalities. Panel bottom-right: we show the top 5% flows of visitors across municipalities excluding Milan. This figure was realized using the R software (4.2.3 version).The R software is available at the following link: https://www.r-project.org/.

Figure 1 shows the top 5% flows across municipalities in the tourists (upper row) and visitors network (lower row) when including (left column) and excluding (right column) the municipality of Milan. We clearly observe that tourists flows tend to connect municipalities characterized by a larger physical distance, whereas visitors flows mainly link neighbour nodes. For instance, when including Milan in the network, the average travel time across the top 5% flows of tourists is equal to 43.15 minutes (median 30.00), while the same figure accounts for 24.05 minutes (median 20.00) in the visitors network.

When we exclude Milan, the average travel time in tourists flows decreases to 33.22 minutes (median 24.00), but is still larger than the same figure in the visitors network, where it reaches 22.33 minutes (median 18.00). This result is consistent with the fact that individuals search for closer places when they move for short same-day trips, while they are willing to travel larger distances in case they decide to spend the night in the target location.

## Methods

**Figure 2:**
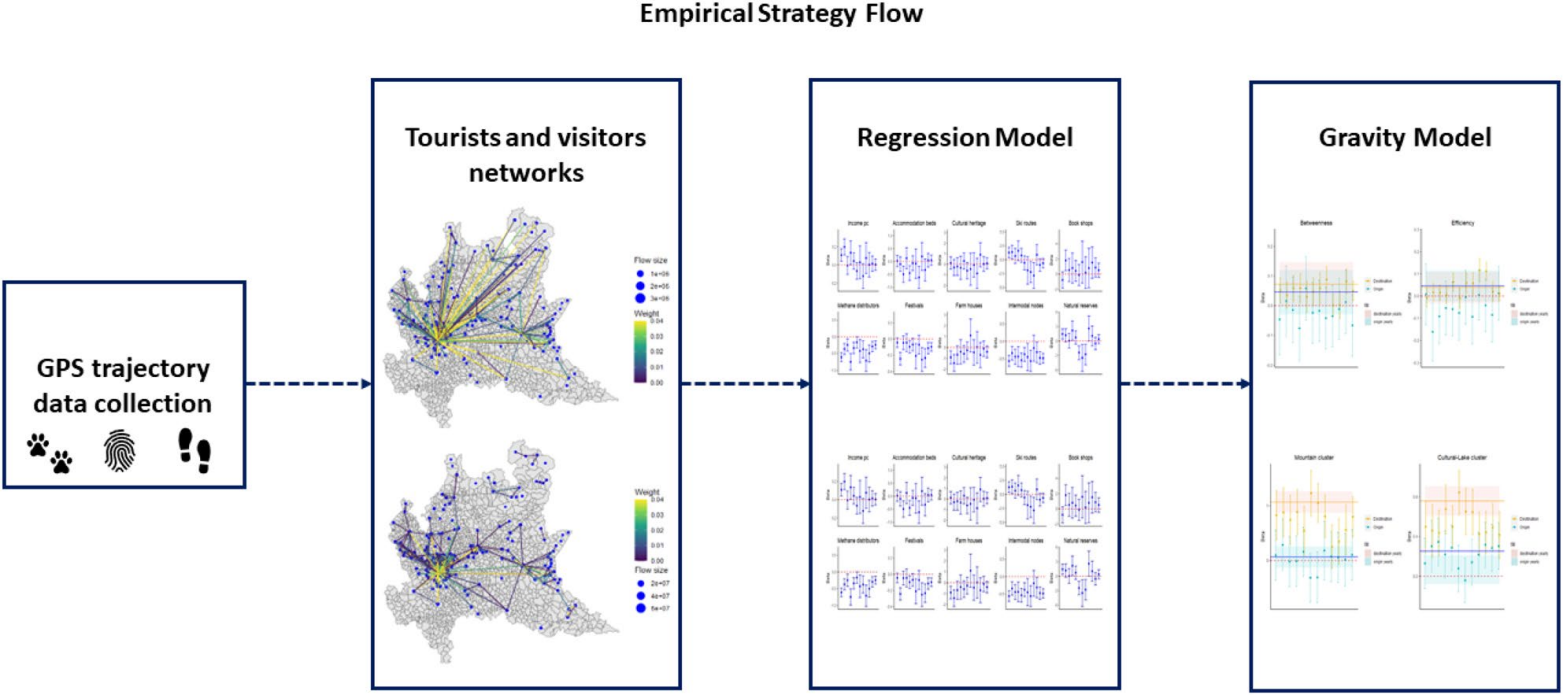
We show the logical flow of our empirical strategy. This figure was realized using Microsoft Power Point.

In this section, we introduce the empirical strategy that we adopt to address our research questions (see Fig. 2).

Section “Centrality variation between tourists and visitors network” introduces the methodology we apply to answer to our *RQ1*. In particular, we describe the regression models we employ to investigate the drivers of the variation of different centrality measures of municipalities in the tourists with respect to the visitors network. In this way, we clarify the main factors contributing to the attractiveness of municipalities in terms of tourists and visitors flows.

In addition, section “Gravity Model” highlights the gravity model used to assess whether centrality indicators in the network of visitors are relevant factors to unveil tourists flows. By the means of this analysis, we aim to discuss our *RQ2*, since we show whether municipalities that receive high tourists flows result to be particularly attractive for visitors, or if instead municipalities tend to receive people with alternative tourism behaviours.

All models described in next Sections are estimated for all months of calendar year 2022. Through the comparison of the results obtained across months, we can thus answer to our *RQ3*, by assessing the stability of identified patterns and the relevance of seasonality factors in explaining tourists flows.

### Centrality variation between tourists and visitors networks

To address our *RQ1*, we analyse the factors mainly fostering tourists and visitors flows. In particular, we investigate the drivers of variation of centrality indicators computed on the network of tourists and visitors. We consider directed weighted networks where municipalities constitute our nodes and the number of people moving between municipalities represent the weight of each edge.

We do this through an OLS model estimated for each month of 2022 to assess the stability of factors influencing tourists and visitors flows along the year and analyse seasonality patterns, as highlighted in our *RQ3*. We rely on the following model:

1 $$\begin{aligned} Y_i = \alpha _0 + \beta *X_i + \epsilon _i \end{aligned}$$

where $Y_i$ is the percentage variation of the same network indicator computed for municipality *i* in the network of tourists and visitors. More in detail, we compute $Y_i$ with the following formula:

2 $$\begin{aligned} Y_i = \frac{(Tourists \ network \ centrality \ indicator - Visitors \ network \ centrality \ indicator)}{Tourists \ network \ centrality \ indicator} \end{aligned}$$

In particular, we focus on instrength, betweenness and efficiency network centrality indicators (see Section SI2 for further details on the computation of such variables). We account for such variables since they provide alternative and complementary information on the role of nodes in the networks. The instrength allows us to study the factors that attract the largest volumes of individuals inflows. Through the betweenness, we evaluate the main features of nodes that are frequently part of shortest paths between other nodes in the network, thus potentially representing bridges between communities of nodes with limited connections among them. Finally, the efficiency enables to analyse the extent to which nodes are close to other municipalities, thus having a critical role to foster people flows within the network.

In terms of regressors, $X_i$ is a vector summarizing the tourism offer in terms of services and attractions provided by each municipality.

We model as dummy variables all those factors that are not present in the majority of analysed municipalities, where the relevant information is thus related to the availability and not to the number of services or tourism attractions. Such binary variables include *Cultural heritage* items, since the presence of cultural and artistic endowments may increase the tourists flows in a place^25,43^. Similarly, natural amenities are considered as factors influencing the attractiveness of locations. For this reason, our model includes the variable *Natural reserves*^44,45^. Furthermore, the presence of leisure and entertainment activities may stimulate additional arrivals in a place. We thus consider the presence of *Ski routes* and *Festivals*^24,46^. As the tourism level is affected by the availability of accommodation infrastructures, we account for the presence of *Farm houses* in each municipality^25,47^. We include the endowments of local transportation infrastructures by considering whether municipalities present some *Intermodal nodes* and are characterized by the presence of *Methane distributors* since they may affect the likelihood that people transit from such nodes during their trip^26,48,49^.

In addition, we consider a set of numerical variables that are related to local economic characteristics or tourism infrastructures and cultural services that are available for the whole sample. In particular, we include the *Income per taxpayer* since local economic conditions may foster people outflows or affect the attractiveness level of municipalities^27,50,51^. Finally, we account for the number of *Accommodation beds* and the number of *Book shops* per inhabitant.

Descriptive statistics on the dependent and independent variables used in this analysis and further details on their sources are available in Section SI3 in Table S2.

### Gravity model

We further explore the relationship between network indicators computed on the network of visitors and tourists. This analysis contributes to answering to our *RQ2* by explaining whether areas receiving high tourists flows are particularly appealing also for visitors, or if instead municipalities tend to attract alternative tourism behaviours. In particular, we aim to evaluate whether tourists flows can be explained by the centrality of municipalities in the network of visitors according to different centrality indicators. Therefore, we employ a gravity model, based on the following equation^52^:

3 $$\begin{aligned} Y_{i,j} = G*Z_{i}^{\beta }*Z_{j}^{\gamma }*dist_{i,j}^{\delta }*\epsilon _{i,j} \end{aligned}$$

The logarithmic version of this model can be estimated through linear models. Therefore, we estimate the following gravity model through a traditional OLS method with a Gaussian error term $\epsilon _{i,j}$.

4 $$\begin{aligned} log(Y_{i,j}) = \alpha _0 + \beta *log(Z_{i}) + \gamma *log(Z_{j}) + \delta *log(dist_{i,j}) + \epsilon _{i,j} \end{aligned}$$

To also address our *RQ3* by assessing seasonality and the stability of drivers of tourism, we estimate the gravity model described in Eq. (4) for each month of year 2022. In particular, $Y_{i,j}$ is the total aggregate monthly flow of tourists moving from municipality *i* to municipality *j*.

$Z_{i}$ is a vector of characteristics of municipalities of origin of the tourists flow. More in detail, it includes the *Income per taxpayer* and the *Population* of the nodes of origin, since a higher wealth and population may foster larger outflows^24,26,43,44^. Moreover, it encompasses a set of network indicators describing the centrality of the underlying municipality in the network of visitors. It includes *Instrength*, *Outstrength*, *Betweenness*, *Authority score*, *Hub score*, and *Efficiency* (see Section SI2 for more details on how we compute such network centrality indicators). Finally, since people flows may be driven by the local characteristics of territories that attract alternative tourism behaviours, we consider a categorical variable, representing the cluster to which each origin municipality is allocated based on a set of social, economic and environmental variables. Specifically, through this categorical variable we distinguish among municipalities in the *Cultural-Lake*, *Mountain* or *Not Specific* tourism cluster. Details on the cluster analysis are provided in Section SI4.

$Z_{j}$ accounts for the same set of factors included in vector $Z_{i}$ but with reference to the municipalities of destination of the tourists flow.

Finally, $dist_{i,j}$ is a vector including alternative measures of distance between node *i* and *j*. It encompasses the travel distance between the municipality of origin and the municipality of destination (The estimation of travel times between all Lombardy’s municipalities is made through r5r, an open-source R package for routing on multi-modal transport networks^53^. This tool is able to run a simulation model, leveraging geographical data about Lombardy’s municipalities locations and road networks, to obtain the travel times by car).

Furthermore, in line with^22^, we consider the availability of tourism services and attractions that are within the *Travel distance* between node *i* and *j*. This is in the spirit of radiation models, suggesting that the distance between areas should be measured not only accounting for geographical distance, but also in terms of density of attractions between the two nodes. In particular, the higher the density of tourism attractions between the two municipalities, the lower the expected flow between such nodes, since people may be attracted by other nodes with a relevant tourism offer between them.

We consider the total number of visits to *Museums* in year 2021, *Cultural heritage* items, *Ski routes*, *Farm houses*, *Intermodal nodes*, *Methane distributors* and *Festivals* in the municipalities with a travel distance from the origin lower than that to travel between nodes *i* and *j*. Each of these variables is multiplied by the average travel distance (from the origin) to reach the nodes in between node *i* and node *j* since such “intermediate nodes” may be more attractive and reduce flows between *i* and *j* in case they are close and easily accessible from the origin of the movement.

Overall, Section SI5 provides additional details on the dependent variable and set of regressors included in the gravity model introduced by Eq. (4). In particular, descriptive statistics of tourists flows, network centrality indicators and on the set of variables describing the availability of tourism services and attractions that are within the travel distance between node *i* and *j* are included in Table S4.

## Empirical evidence

### The drivers of centrality variation in tourists and visitors networks

In this section, we show the results related to our *RQ1*, through the analysis of the main factors influencing the variation of centrality of municipalities in the tourists network with respect to the visitors network. In particular, we focus on drivers of variation of instrength, betweenness and efficiency over the 12 months of 2022. This analysis may thus support policy makers in a better comprehension of the main factors stimulating alternative types of tourism behaviour.

**Figure 3:**
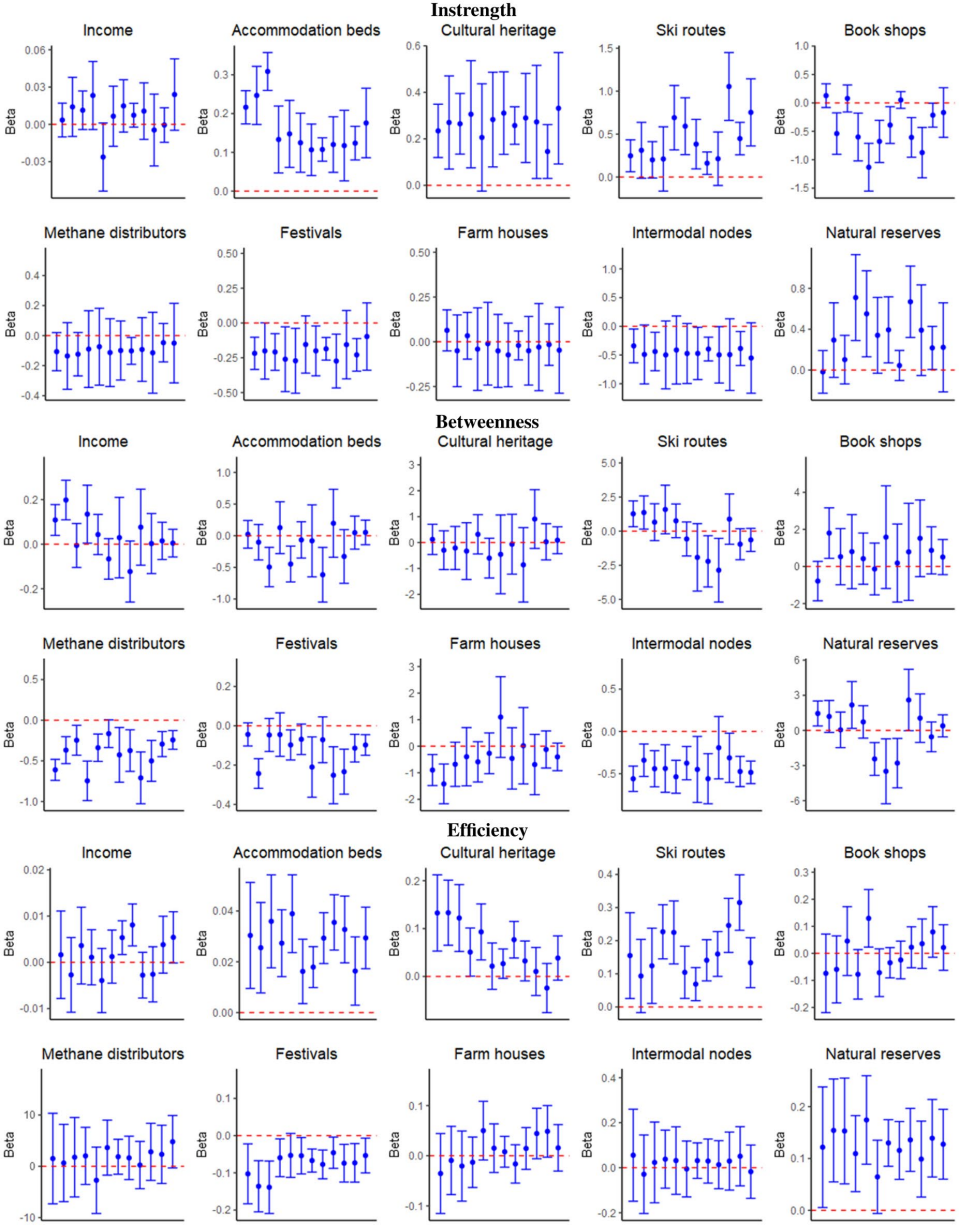
We show the monthly coefficients of drivers of delta performances of a set of centrality indicators computed in the tourists and visitors networks. The plots refer to the estimates obtained through the model introduced in Eq. (1). The upper panel refers to drivers of instrength variation. The middle panel refers to drivers of betweenness variation. The lower panel refers to drivers of efficiency variation. This figure was realized using the R software (4.2.3 version).

In Fig. 3, the upper panel shows the coefficients of a set of variables related to tourism services and attractions aiming to explain the variation of instrength in the tourists with respect to the visitors network. Interestingly, one additional accommodation bed induces higher tourists inflows rather than visitors trips between 0.11% and 0.31%. The positive relationship is stable along the year, with a higher magnitude in the first three months of the years.

Furthermore, the presence of cultural heritage items and ski routes tends to increase the centrality of nodes in the tourists with respect to the visitors network in terms of instrength by a portion between 0.15% and 0.33% and between 0.16% and 1.05%, respectively. During Spring and Summer months, with the exception of August, also natural reserves contribute to raising tourists with respect to visitors inflows ($\beta$ between − 0.01 and 0.71). On the other hand, festivals ($\beta$ between − 0.27 and − 0.09) and the presence of intermodal nodes ($\beta$ between − 0.55 and − 0.34) reduce the instrength centrality in the tourist network, as well as the book shops ($\beta$ between − 1.13 and 0.12) with the exception of January, March, August and December.

Such results suggest that the availability of tourism accommodation infrastructures, natural and cultural endowments and services related to ski activities foster overnight stays rather than short visits. On the other hand, temporary entertainment activities (e.g. festivals) or transportation infrastructures, such as intermodal nodes, boost short term visits with respect to tourists inflows.

The middle panel in Fig. 3 analyses the drivers of the percentage variation of betweenness in the tourists with respect to the visitors network. Interestingly, we observe a different pattern with respect to that observed for the instrength. Indeed, in this case accommodation infrastructures, cultural and natural attractions do not foster this centrality indicator in the tourists network. On the other hand, we highlight that for the betweenness, critical factors are represented by transportation services and infrastructures. Indeed, we identify a negative and statistically significant coefficient along the whole year for methane distributors ($\beta$ between − 0.75 and − 0.16) and intermodal nodes ($\beta$ between − 0.56 and − 0.19), thus meaning that higher values of these variables stimulate higher betweenness in the visitors network with respect to the tourists network by percentages between 0.16% and 0.75% and between 0.19% and 0.56%, respectively.

This result suggests that nodes with higher transportation infrastructures endowments tend to be present in many shortest paths connecting municipalities in the visitors network, meaning that they are key factors to attract short term visits, while they do not represent characteristics stimulating strong tourists flows. We find a similar negative coefficient also for festivals ($\beta$ between -0.27 and -0.05), confirming that such temporary recreational activities may foster short term visits with respect to overnight stays by a percentage between 0.05% and 0.27%.

We observe that the availability of ski routes increases the betweenness in the tourists network during winter months (e.g. January, February), while it raises short term visits in the summer months of August and September, thus highlighting the role of skiing activities in attracting alternative types of tourism behaviours, depending on whether the “snow season” is open or not.

The lower panel of Fig. 3 investigates the factors that contribute to a variation of efficiency in the tourists with respect to the visitors network.

Similarly to the instrength, we find that a larger availability of accommodation beds ($\beta$ between 0.02 and 0.04), ski routes ($\beta$ between 0.07 and 0.31) and natural reserves ($\beta$ between 0.06 and 0.17) increases this centrality indicator for the tourists network.

Also the presence of cultural heritage items ($\beta$ between − 0.02 and 0.13) raises the proximity of municipalities to the other nodes in the tourists network, especially in the first half of the year. We rather confirm that festivals ($\beta$ between − 0.14 and − 0.05) are key drivers of visitors flows. Differently from the instrength case, we do not find a negative impact of intermodal nodes, suggesting that transportation infrastructures do not reduce the centrality of nodes in the tourists with respect to the visitors network. This may be due to the fact that the instrength considers only individuals inflows, whereas the efficiency accounts for both people exiting from and entering into a municipality.

Overall, such results highlight the heterogeneity of drivers of tourists and visitors movements. In particular, this analysis, related to our *RQ1*, shows that the former tend to be more attracted by the availability of accommodation infrastructures, natural and cultural endowments, while transportation services and temporary entertainment activities mainly foster same-day visits.

Moreover, we find that some attractions such as ski routes and natural reserves may further stimulate tourists flows during Spring/Summer and Winter, respectively, thus suggesting the relevance of seasonal patterns, as questioned by our *RQ3*. This evidence may thus allow policy makers to identify the main leverages to design more precise strategies to attract specific types of tourism behaviour.

### Gravity model evidence

Since Lombardy municipalities display relevant percentage variations in terms of instrength, betweenness and efficiency computed on the network of visitors and tourists, we investigate the extent to which centrality indicators based on same-day visits might be relevant drivers of overnight stays flows. We do this through the gravity model introduced in Eq. (4). This analysis aims to address our *RQ2* and to study the extent to which areas attracting high levels of tourists are particularly appealing also for visitors, or if instead municipalities tend to attract people with alternative tourism behaviours.

**Figure 4:**
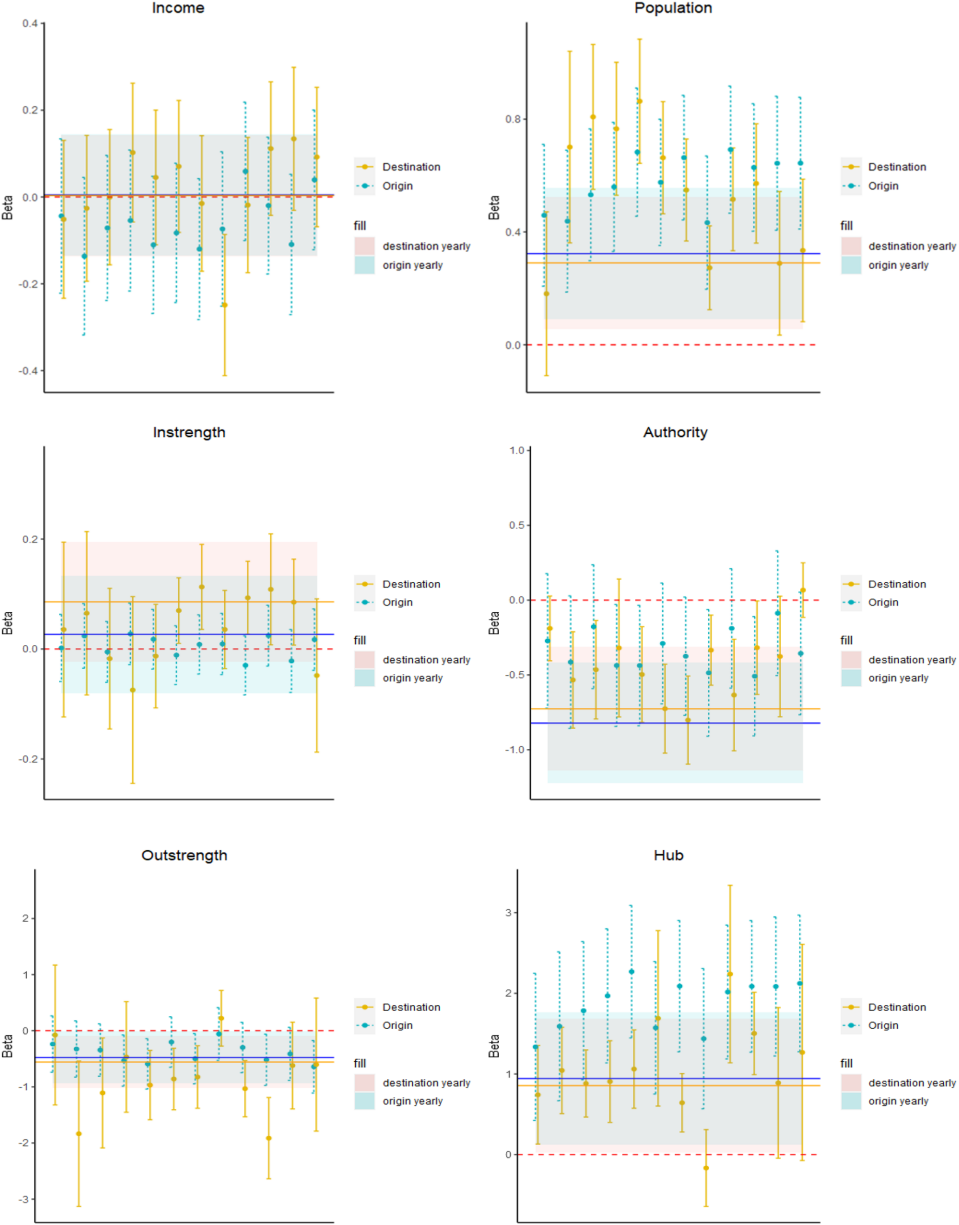
The monthly coefficients of drivers of monthly flows across municipalities in Lombardy. The plots refer to the estimates obtained through the gravity model introduced in Eq. (4). Vertical segments refer to the point estimates with 95% confidence intervals estimated over the 12 months. The horizontal line refers to the point estimates and 95% confidence intervals of the same gravity model using aggregate yearly (rather than monthly) tourists flows across Lombardy municipalities. Part I. This figure was realized using the R software (4.2.3 version).

**Figure 5:**
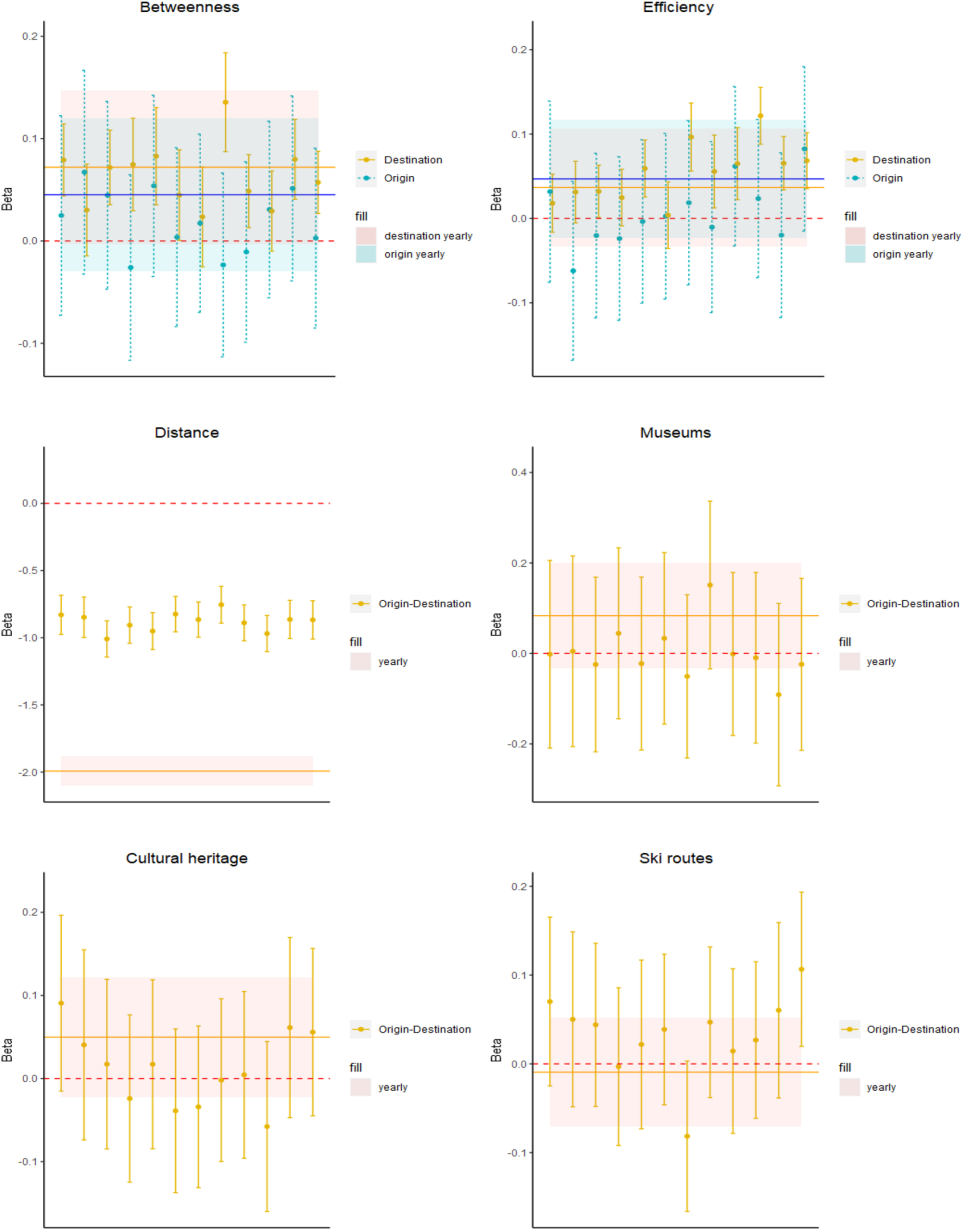
The monthly coefficients of drivers of monthly flows across municipalities in Lombardy. The plots refer to the estimates obtained through the gravity model introduced in Eq. (4). Vertical segments refer to the point estimates with 95% confidence intervals estimated over the 12 months. The horizontal line refers to the point estimates and 95% confidence intervals of the same gravity model using aggregate yearly (rather than monthly) tourists flows across Lombardy municipalities. Part II. This figure was realized using the R software (4.2.3 version).

**Figure 6:**
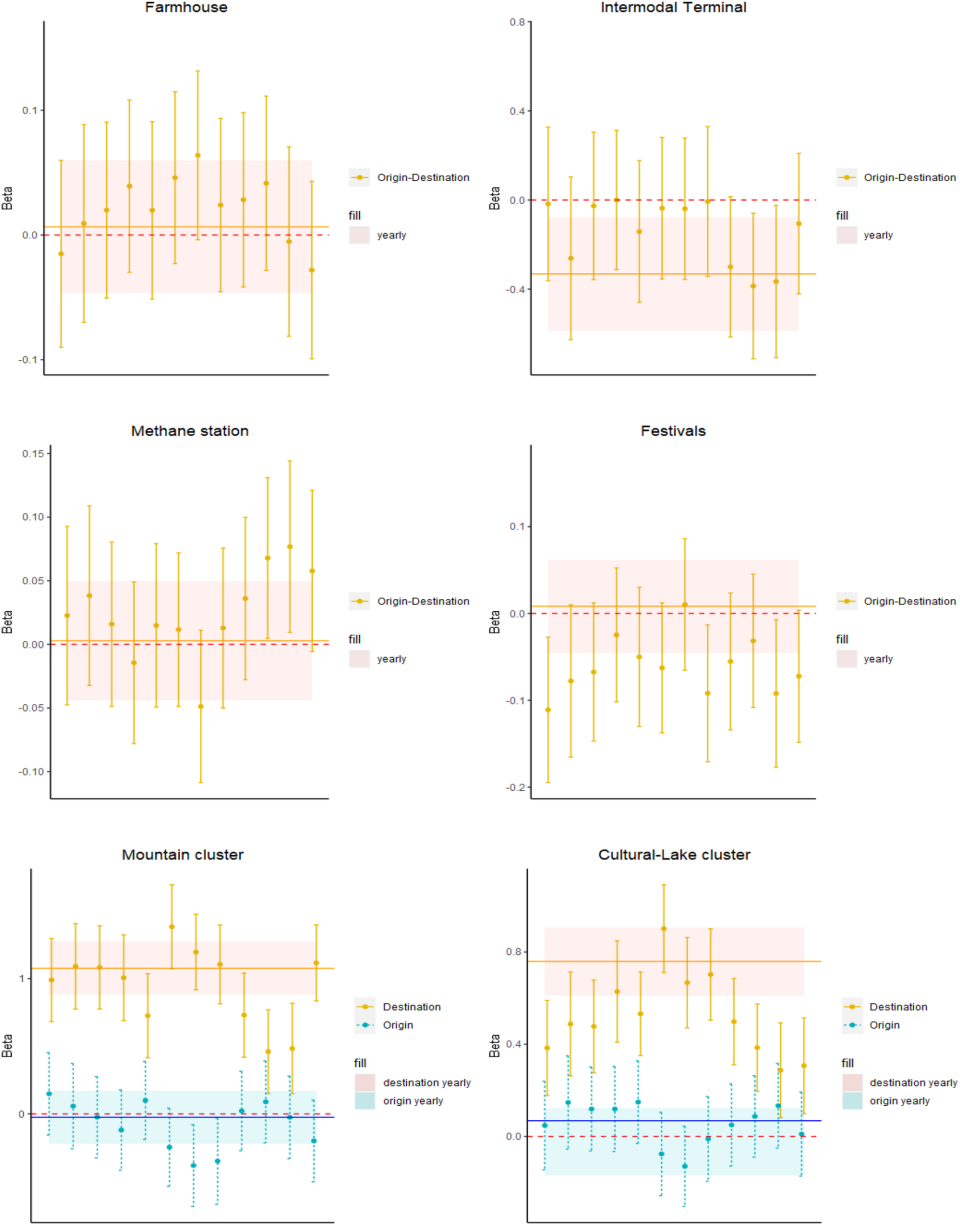
The monthly coefficients of drivers of monthly flows across municipalities in Lombardy. The plots refer to the estimates obtained through the gravity model introduced in Eq. (4). Vertical segments refer to the point estimates with 95% confidence intervals estimated over the 12 months. The horizontal line refers to the point estimates and 95% confidence intervals of the same gravity model using aggregate yearly (rather than monthly) tourists flows across Lombardy municipalities. Part III. This figure was realized using the R software (4.2.3 version).

Figures 4, 5 and 6 show the estimates of the coefficients of the main drivers of tourists flows across Lombardy municipalities over 12 months of 2022 with related confidence intervals (see Section SI5 and Tables S5, S6, S7 and S8 for more details). Furthermore, we assess the corresponding coefficient obtained through a model aggregating yearly flows across municipalities. In this way, in line with our *RQ3*, we assess whether coefficients of this set of variables are stable over the year or seasonal patterns strongly affect the main factors stimulating tourism.

Consistently with gravity model theory, we observe the stronger flows of tourists are observed between municipalities with a larger number of inhabitants in line with previous results obtained by^24^ and^25^ for the Italian context. According to the yearly model, a 1% growth of origin municipality population leads to an increase of tourists flows equal to 0.32%. The same figure accounts for 0.30% for destination municipalities. Coefficients with a stronger magnitude and statistically significant are observed when we consider monthly models, with values between 0.44 and 0.69 for origin places and in the range 0.18–0.86 for destinations (the only exception is the month of January where the coefficient is still positive but not statistically significant).

Interestingly, we find a not statistically significant estimate for the instrength of destination municipalities over the first half of the year (some exceptions are June, July, September, October and November in the second half of the year). This result suggests that at least over the period January-May, places with large tourists inflows are not necessarily characterized also by a large number of visitors, highlighting the presence of different drivers in play for overnight stays and same-day trips. This result is reinforced by the fact that the coefficient for the yearly model is positive but not statistically significant. On the other hand, more similar determinants between tourists and visitors flows seem to be in place during the Summer and Autumn months.

Such evidence is complemented by the authority score of destinations that tends to exhibit a negative and significant coefficient ($\beta$ between − 0.81 and − 0.19) with few exceptions (April, December). This is particularly interesting since it can be interpreted as if municipalities with strong tourism inflows are receiving limited visitors flows from hubs in the visitors network. Therefore, the combined evidence of instrength and authority variables directly addresses our *RQ2*, by suggesting that municipalities with large tourists inflows are not necessarily characterized by a large number of same-day visits. Furthermore, such visitors do not come from municipalities that experience large visitors outflows. This evidence also holds at aggregate annual level with a significant $\beta$ = − 0.68. Such result corroborates that alternative drivers may motivate tourists and visitors flows, meaning that, for policy makers, it may be complex to attract at the same time alternative types of tourism behaviours.

We observe that the instrength of origin municipalities has a not statistically significant coefficient over the whole year. This means that the number of tourists outflows is not strongly related to the number of visitors inflows. Such relationship is quite stable across months of 2022 and confirmed in the aggregate yearly model. This points to the fact that over the whole year, large tourists outflows are not necessarily compensated by high visitors inflows. Conversely, we obtain a negative coefficient for the authority of origin places at annual level ($\beta$ = -0.82). Such relationship, that is confirmed at monthly level with a lower statistical significance, suggests that in municipalities with large tourists outflows, visitors inflows do not come from municipality hubs in the visitors network.

We find a different result for the outstrength indicator. For the origin municipalities the coefficient is negative and statistically significant over a significant portion of the year (e.g. in the months of April, May, July, October, November and December with values between − 0.63 and − 0.41). This pattern shows that places with large outflows of tourists are characterized by a not significant or even negative relationship with the volume of visitors exiting from the same municipality, suggesting that territories tend to experience outflows of individuals with alternative tourism behaviours. The coefficient of the yearly model is negative and statistically significant ($\beta$ = − 0.48), meaning that the different patterns of tourists and visitors outflows in the same municipality are stable along the year.

Also in this case such result can be complemented by the coefficient obtained for the hub score of origin municipalities to address our *RQ2*. The positive and significant ($\beta$ between 1.34 and 2.28) coefficients suggest that territories with large tourists outflows are hubs of the visitors network, meaning that they are municipalities sending visitors towards areas attracting large visitors flows. The combined evidence of outstrength and hub score highlights that places with strong outflows of tourists experience limited visitors outflows. However, those visitors exiting the municipality tend to converge toward areas attracting large short visits inflows.

Similar evidence holds for places of destination for both outstrength and hub score, thus suggesting that areas attracting larger tourist inflows experience a low number of visitors outflows that tend to be catalyzed by municipalities with large numbers of same-day visits.

Combining the interpretation of alternative network indicators, we can thus obtain complementary knowledge on the behaviour of tourists and visitors, supporting the identification of more effective tourism management strategies. For instance, being aware of more likely origins and destinations of tourists and visitors flows may allow policy makers to invest on better connections, transportation facilities, customized services and attractions, improving the economic impact of tourism related activities.

We spot a positive interplay over the majority of months of the year for the betweenness of places of destination ($\beta$ between 0.04 and 0.13 and not significant in the months of February, July and October). Such relationship is confirmed at annual aggregate level ($\beta$ = 0.07 and significant with a confidence level of 10%). This provides evidence that places with large tourists inflows represent nodes belonging to a large number of shortest paths across municipalities in the visitors network. This is particularly relevant since it suggests that nodes bridging alternative communities of areas in the visitors network may be selected also as strategic places to spend the night by tourists.

We rather find a not statistically significant relationship for the betweenness of places of origin. A consistent result is obtained for the efficiency indicator. Also in this case, we find a positive coefficient for places of destination, with such relationship becoming significant especially during the second half of the year ($\beta$ between 0.06 and 0.12). This result corroborates the previous evidence obtained with the betweenness, confirming that large tourists inflows are experienced by municipalities with a key role in spreading visitors mobility. We do not find a statistically significant relationship for places of origin both at monthly and yearly level.

Overall, these relationships between tourists flows and the majority of centrality indicators computed in the network of visitors suggest that alternative drivers may have a critical role in stimulating overnight stays and same-day trips, since areas with large tourists flows are not necessarily central in the visitors network. In terms of our *RQ2*, we highlight the presence of a trade-off in the capability of municipalities to attract at the same time large visitors and tourists flows (In terms of external validity, we should acknowledge that the Lombardy region has some specific peculiarities within the Italian context, since it is the largest in terms of population (accounting for almost 17% of total Italian inhabitants) and the richest in terms of income per capita. Moreover, Milan attracts large mobility flows also due to commuting patterns. Therefore, strong attentions should be provided when trying to generalize such results to other Italian contexts. However, it should be noted that^41^ demonstrated the adequate capability of this mobile network dataset to properly capture tourism related mobility also in the city of Rome, that similarly to Milan, is characterized by a large presence of commuters. Therefore, although findings should be carefully checked depending on the specific territory that is object of analysis, our empirical framework and the dataset may be reasonably applied across different Italian areas to study the main drivers of tourists and visitors flows).

In coherence with the gravity model, we also observe a negative and statistically significant coefficient for the travel distance across municipalities ($\beta$ between − 1.01 and − 0.76), suggesting that stronger tourists flows are observed across municipalities that are closer to each other. Our results confirm previous evidence obtained by^24^ estimating a spatial Durbin model to assess the drivers of international tourism towards Italian provinces. On the other hand, we do not find evidence that a higher density of tourism attractions between municipalities, tends to reduce the flows across such nodes. Few exceptions at a significance level of 10% are the presence of festivals in January, August, November and December, the presence of ski routes in July and intermodal nodes in September October and November.

We rather spot interesting patterns for categorical variables related to the cluster assigned to Lombardy municipalities in Section SI4, and allowing us to better investigate our *RQ3*.

Our reference cluster is represented by the *Not specific* tourism group. We observe that destinations in the *Mountain* cluster experience larger tourists flows along the year ($\beta$ between 0.45 and 1.38), with coefficient that are higher during Winter months (e.g. December-March) when people have the opportunity to ski or in the Summer (e.g. June, July, August), in a period characterized by milder climate conditions.

Interestingly, also *Cultural-Lake* areas exhibit larger tourists inflows, as suggested by the positive coefficients for destination places. In this case, the coefficient is higher during Summer, whereas it is lower during Winter months, suggesting that tourists prefer spending time in such places in periods characterized by better weather conditions ($\beta$ between 0.29 and 0.90). In both cases, we rather do not tend to find evidence of significant coefficients for places of origin, suggesting that drivers to exit the municipality of residence for tourism reasons are homogeneous across clusters over the year.

Concerning our *RQ3*, the variability of estimated coefficients across different months of the year highlights the need to rely on high frequency data, enabling to account for seasonality patterns to support policy makers taking informed decisions based on robust data driven evidence.

## Conclusions

In this paper we exploit mobile phone network data to study two alternative types of tourism behaviour: overnight tourists and same-day visitors. We contribute to the extant literature analysing the main factors influencing people length of stay at a destination, whilst the majority of current studies considers only overnight stays, completely neglecting same-day visits.

Our analysis first aims to identify the main determinants of the attractiveness level of Lombardy municipalities, disentangling between tourists and visitors flows. Second, we point to discuss the extent to which places receiving high tourists arrivals result particularly appealing also in terms of visitors. Finally, we highlight whether such patterns are characterized by relevant seasonality or they are stable along the year.

Concerning the first point, we demonstrate that municipalities offering superior tourist services and attractions, including accommodation options, cultural heritage sites, ski routes, and natural reserves, tend to have higher tourists inflows. Conversely, temporary entertainment activities such as festivals and the availability of transportation facilities (e.g. methane stations and intermodal nodes) raise areas attractiveness especially for same-day visits.

As regards our second research purpose, the different drivers fostering tourists and visitors flows highlight the presence of a trade-off in the capability of municipalities to attract at the same time overnight stays and one-day visits. We confirm such hypothesis, observing that higher numbers of tourists inflows are exhibited by areas not necessarily receiving large levels of visitors. Similarly, we find that places characterized by large tourists outflows display limited volumes of individuals visiting for a one day trip other places. In this sense, limited exceptions are represented by municipalities with a key role in spreading the mobility in the visitors network, representing nodes that constitute a bridge between communities of municipalities displaying limited connections among them. These places, exhibiting large values of betweenness, account for high flows both in terms of tourists and visitors.

Finally, we demonstrate that mobile phone network data display a high level of temporal and spatial granularity, thus representing an adequate source of information to support the design of tourism management strategies based on real world evidence. We highlight that alternative tourism classes might experience different levels of attractiveness along the year with relevant seasonality patterns. For instance, *Mountain* areas experience larger visits when ski facilities are open or during Summer, probably due to better weather conditions. On the other hand, *Cultural-Lake* territories display higher tourists flows during Spring and Summer, corroborating that climate conditions are also relevant drivers of tourism.

Overall, our main findings can have relevant implications for policy makers highlighting how mobile network data can support their decision making processes. Our results show how these innovative data sources have a spatial and time resolution that make them a valid tool for the identification of the specific drivers of alternative touristic behaviours, thus supporting policy makers in the definition of precision policies in the tourism sector. In other words, these data allow to design tailored policies that can strengthen tourists’ flows leveraging on the peculiar characteristics of each territory. For instance, receiving at the same time tourists and visitors flows is inherently complex; hence, tourism management strategies may be more effective in case policy makers first comprehend the target tourism behaviour they want to attract in their territory, and then design a coherent strategy in terms of services, attractions, and recreational activities. In this direction, our analysis may enable the design of clusters of tourist destinations that are geographically close and characterized by complementary tourism related services. These destinations could develop integrated strategies by identifying a common hub, where accommodation infrastructure and cultural services attracting tourists could be located, while dedicating surrounding areas to destinations for visitors with entertaining and recreational activities. This approach would avoid potential resources duplication enhancing territorial synergies. These findings are even more relevant for policy makers to properly manage the expected growth in tourism related flows in next years due to the Winter Olympic Games “Milano-Cortina 2026”.

Despite our effort to implement methodologically grounded research, some limitations still affect this work and may open future research opportunities and discussion. First, our data are disclosed by a relevant telecommunication company but are not provided by national statistical offices. Therefore, rumor and imprecision might be present in our original data, although they have a good match with official statistics. Moreover, other limitations affect mobile network data. For instance, they are normally disclosed by telecommunication companies under specific contracts and agreements with policy makers and researchers, but are not normally available to the wide community, thus significantly restricting the number of people that could extract value from them. Moreover, they need additional validation checks ensuring that they actually capture tourism related flows and not just mobility patterns related to commuting. Finally, they lack complementary information related that are usually associated to questionnaire-based surveys, such as the reason for the travel.

Furthermore, we cover only 163 municipalities in Lombardy. Despite the high representativeness of such tourists flows, future studies may try to include a larger portion of municipalities to assess whether our findings hold on a larger set of territories. Finally, we only focus on the time frame January-December 2022. Next steps of our work might be connected with an extension of the analysed period to evaluate the robustness of our results also on a different calendar year.

### Supplementary Information

Supplementary Information.

## Data Availability

Data describing the visitors and tourists flows across Lombardy municipalities have been provided by Motion Analytica leveraging on mobile network data gathered by Vodafone Business Italia and made available to the authors by Polis, the institute supporting policy design of Regione Lombardia, the regional government of Lombardy. All social, economic, demographic and environmental variables related to the Lombardy municipalities have been collected from the Italian National Institute of Statistics (ISTAT, http://dati.istat.it/) or from the Open Data Lombardia platform (https://www.dati.lombardia.it/).
