# Supplementary material for: Exploring drivers of overnight stays and same-day visits in the tourism sector
Source: Sci Rep. 2024 Apr 29;14:9840. doi: 10.1038/s41598-024-60229-w (PMC11058821; doi:10.1038/s41598-024-60229-w)
Supplement: Supplementary file 1 — Supplementary Information. [file 41598_2024_60229_MOESM1_ESM.pdf]

Supplementary Information for: Exploring drivers of overnight stays and same-day visits in the tourism sector

Francesco Scotti[1,2], Andrea Flori[1,2], Piercesare Secchi[3], Marika Arena[1,2], and Giovanni Azzone[1,2]

[1] *Department of Management, Economics and Industrial Engineering, Politecnico di Milano, Via Lambruschini, 4/B, 20156, Milan, Italy.*
[2] *Impact, Department of Management, Economics and Industrial Engineering, Politecnico di Milano*
[3] *MOX - Dipartimento di Matematica, Politecnico di Milano, Piazza Leonardo da Vinci 32, 20133 Milano, Italy*
[*] *corresponding author: francesco.scotti@polimi.it*

ABSTRACT

This paper aims to explore the factors stimulating different tourism behaviours, with specific reference to same-day visits and overnight stays. To this aim, we employ mobile network data referred to the area of Lombardy. The paper highlights that larger availability of tourism accommodations, cultural and natural endowments are relevant factors explaining overnight stays. Conversely, temporary entertainment and transportation facilities increase municipalities attractiveness for same-day visits. The results also highlight a trade-off in the capability of municipalities of being attractive in connection to both the tourism behaviours, with higher overnight stays in areas with more limited same-day visits. Mobile data offer a spatial and temporal granularity allowing to detect relevant patterns and support the design of tourism precision policies.

1 Data Validation

Figure 1 shows the geographical distribution of the set of 163 municipalities included in our analysis. Furthermore, Table 1 reports the name of the 163 municipalities that are analysed in our paper.

Table 1. We list the name of the 163 municipalities that are included in our analysis.

Agrate Brianza	Ferno	Ponte San Pietro
Albavilla	Gallarate	Ponti Sul Mincio
Aprica	Garbagnate Milanese	Porlezza
Arcore	Garbagnate Monastero	Pozzolengo
Assago	Gardone Riviera	Rezzato
Bagnatica	Gargnano	Rho
Bagolino	Grandate	Riva Di Solto
Baranzate	Grassobbio	Rodengo Saiano
Basiglio	Gravedona Ed Uniti	Rozzano
Bergamo	Guardamiglio	Salo
Binasco	Idro	San Donato Milanese
Bollate	Iseo	San Felice Del Benaco
Borgo Virgilio	Lainate	San Giorgio Bigarello
Bormio	Laveno-Mombello	San Martino In Strada
Brembate	Lecco	San Pellegrino Terme
Brescia	Legnano	San Siro
Bresso	Lezzeno	San Vittore Olona
Busto Arsizio	Limbiate	Sarnico
Cambiago	Limone Sul Garda	Saronno
Campodolcino	Lissone	Segrate
Cardano Al Campo	Livigno	Sesto Calende
Carpiano	Lodi	Sesto San Giovanni
Casalmaggiore	Lomazzo	Settala
Caspoggio	Lonato Del Garda	Settimo Milanese
Cassano D'Adda	Lovere	Sirmione
Castenedolo	Luino	Soiano Del Lago
Castiglione Delle Stiviere	Maccagno Con Pino E Veddasca	Solbiate Olona
Castione Della Presolana	Madesimo	Somma Lombardo
Cavenago Di Brianza	Malgrate	Sondrio
Centro Valle Intelvi	Mandello Del Lario	Stezzano
Cernusco Sul Naviglio	Manerba Del Garda	Teglio
Cerro Maggiore	Mantova	Temu
Cesano Maderno	Marone	Tignale
Chiavenna	Menaggio	Tirano
Chiesa In Valmalenco	Milano	Toscolano-Maderno
Cinisello Balsamo	Moniga Del Garda	Tremosine Sul Garda
Clusone	Montano Lucino	Trezzano Sul Naviglio
Colico	Montichiari	Turate
Cologno Monzese	Monza	Val Masino
Como	Monzambano	Valbondione
Concorezzo	Mozzo	Valdidentro
Corbetta	Novate Milanese	Valdisotto
Cornaredo	Novedrate	Valfurva
Corteno Golgi	Olgiate Olona	Varedo
Crema	Orio Al Serio	Varese
Cremona	Orzivecchi	Verdellino
Cusago	Ospedaletto Lodigiano	Vergiate
Dalmine	Ossona	Vermiglio
Darfo Boario Terme	Padenghe Sul Garda	Vezza D'Oglio
Dervio	Paderno Dugnano	Vigevano
Desenzano Del Garda	Pavia	Vimercate
Desio	Pero	Vione
Dongo	Peschiera Borromeo	
Erba	Pieve Emanuele	
Erbusco	Ponte Di Legno	

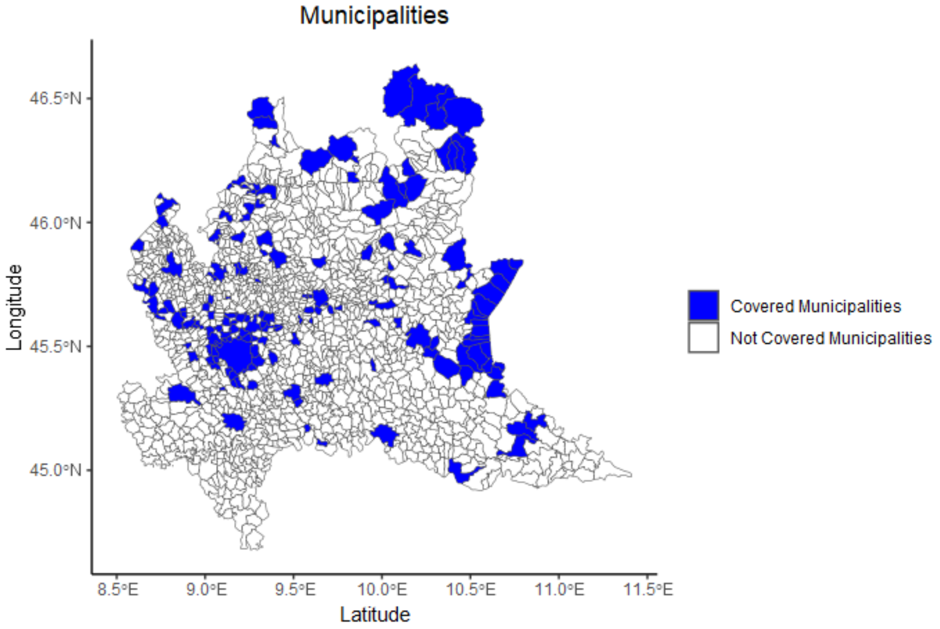

Figure 1. The geographical distribution of the 163 municipalities covered by our empirical analysis. This figure was realized using the R software (4.2.3 version).

The mobile network data used in this paper have been made available to the authors by Polis, a public entity collaborating with Lombardy region. Consequently, we first check the extent to which they are representative of tourists flows in Italy, by comparing them against information disclosed by the national statistical office (ISTAT).

As a first step, we match the number of tourists presences in our mobile network data and ISTAT dataset. In particular, we compare our data, available only in 2022, with official statistics provided by ISTAT for the years 2019, 2020 and 2021 (the three most recent years at the moment of writing). Figure 2 highlights the high correlation between ISTAT and our mobile network data with coefficients ranging between 0.92 and 0.97 (p-values ~ 0 in all cases), thus suggesting that we properly capture tourists dynamics (the same figures are 0.61, 0.53 and 0.62 with p-values ~ 0 in all cases, when we exclude Milan from our sample).

To further investigate the extent to which our data are representative of tourists flows in Lombardy, we compute the portion of tourists presences covered by the 163 municipalities included in our analysis with respect to total tourists presences in the Lombardy region. To do this, we exploit ISTAT data that cover the entire set of Lombardy municipalities. Figure 3 highlights that the 163 municipalities included in our dataset cover the main tourism areas in Lombardy, accounting for percentages equal to 0.88, 0.86 and 0.85 in 2019, 2020, and 2021 of total tourists presences in Lombardy.[1]

We then check the capability of our mobile network data to properly capture seasonality and time patterns along the year in the tourists flows.

We thus compute for each month, the aggregate number of presences observed in our mobile network and ISTAT data with reference to the same set of 163 municipalities included in our analysis. Correlations between the two sets of data are equal to 0.83 (p-value = 0.001), 0.02 (p-value = 0.96), 0.68 (p-value = 0.01) in years 2019, 2020, 2021. It is worthy of noticing that, despite the short available time series, the correlation is significant in year 2019, whereas it is not statistically relevant in year 2020, when tourists presences were disrupted by restrictions against the COVID-19 pandemic.

Correlation exhibits a weak statistical significance also in 2021, highlighting a not yet complete recovery of tourists flows. Indeed, restrictions still affected the winter season in 2021, with limitations related to skiing activities. Such results are consistent with the fact that our mobile network data refer to year 2022, when restrictions to tourists flows were not in place.

Overall, we observe larger values in the mobile network dataset with respect to data disclosed by the national statistical office. This result confirms the evidence provided by[1]. In particular, they explain how mobile network data may detect larger tourists flows due to specific factors such as people flows to family/friends, movements towards houses non representing the place of residence, presences in night owls (nightclubs, parties), leisure events (weddings, festivals, sport competitions), movements related to the medical sector (hospitals, convalescence) and due to work reasons (e.g., truckers/transporters).

[1] Such figures are computed as the ratio between the total number of presences of tourists in the 163 municipalities that are included in our analysis (due to the availability of our mobile network data) and the total number of presences of tourists in all Lombardy municipalities. Both the numerator and the denominator are computed based on ISTAT data.

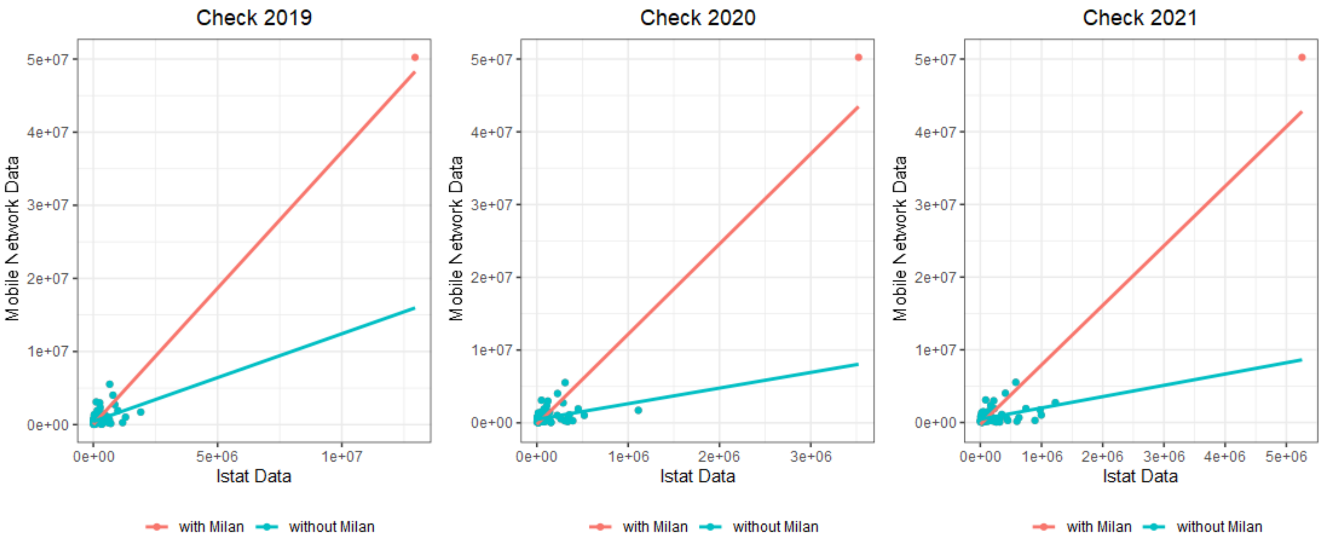

Figure 2. Correlation between the number of tourists presences in our mobile network and ISTAT data. ISTAT data refer to years 2019, 2020 and 2021. Our mobile network data are related to 2022. In case we consider the whole dataset (including the municipality of Milan) correlation coefficients ρ are equal to 0.97, 0.92 and 0.92 when ISTAT data refer to 2019, 2020 and 2021, respectively (see the light red line). In case we exclude the municipality of Milan the same figures account for 0.61, 0.53 and 0.62, respectively (see the light blue line). In all cases p-values$\sim$ 0. Notice how the correlation coefficient is slightly lower in the years affected by the COVID-19 pandemic (that is not captured by mobile network data referring to 2022). This figure was realized using the R software (4.2.3 version).

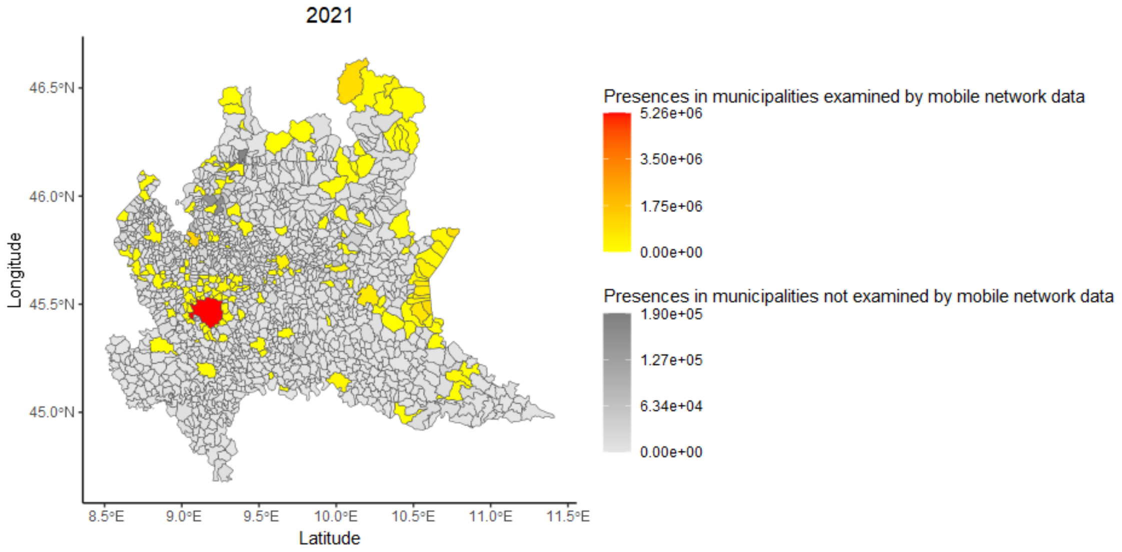

Figure 3. We show tourist presences in the 163 municipalities included in our analysis in a yellow-red scale of colours. We also show tourist presences in the other municipalities not included (due to the unavailability of our mobile network data) in our analysis in a grey scale of colours. Darker colors refer to larger presence volumes. All data (both those underlying the yellow-red scale and those underlying the grey scale) are disclosed by ISTAT and refer 2021. Similar results hold in 2019 and 2020. This figure was realized using the R software (4.2.3 version).

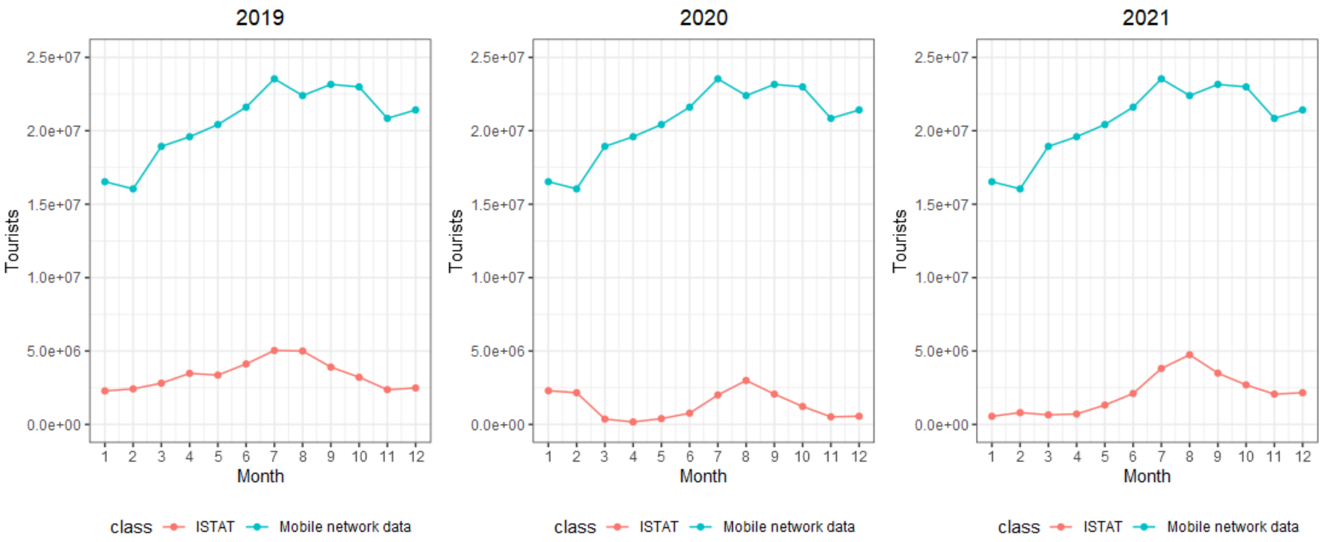

Figure 4. We show a line plot representing aggregate number of presences observed in our mobile network and ISTAT data with reference to the same set of 163 municipalities included in our analysis. Correlation is equal to 0.83 (p-value = 0.001), 0.02 (p-value = 0.96), 0.68 (p-value = 0.01) in years 2019, 2020, 2021. Mobile network data are always related to year 2022. This figure was realized using the R software (4.2.3 version).

As a final step, we asses the extent to which our data complement information related to commuters mobility. Indeed, our mobile network dataset should capture movements related to tourism activities and should not be significantly affected by people who move only for work reasons.

For doing this, we compare total visitors and tourists flows among each couple of analysed municipalities in our dataset with those observed in the origin-destination (OD) matrix disclosed by Lombardy region and referring to year 2020 (most recent year at the moment of writing).[2] We first compute the Pearson correlation coefficient between total visitors and tourists flows in our dataset and the corresponding figure in the OD 2020 matrix. Overall, we obtain a ρ equal to 0.47 (p-value $\sim$ 0) and 0.27 (p-value $\sim$ 0) for visitors and tourists. Although the correlation is statistical significant, notice how these values point to the fact that our mobile network dataset is providing complementary information with respect to commuters mobility. Furthermore, it is reasonable to obtain a larger correlation for visitors, since same-day visits normally involve places at limited distance where people can also move for daily work purposes.

Finally, we build the adjacency matrix W of people flows between each couple of municipality i and j (e.g., $w_{i,j}$ is the number of individuals moving from node i to node j). We also standardize each cell with respect to the row sum, such that each cell represent the percentage of people travelling from municipality i to municipality j with respect to the total number of people exiting from node i. Figure 5 show the absolute value of differences across these row standardized adjacency matrices in case we consider tourists or visitors flows with respect to commuters flows obtained in the OD 2020 matrix. We show that differences are distributed across municipalities with larger values concerning the municipality of Milan. This is probably due to the high volume of commuters attracted by Milan that tend to be larger than tourists or visitors reaching Milan.

[2]The dataset with the OD matrix for Lombardy region for year 2020 is available at the following link: `https://www.dati.lombardia.it/Mobilit-e-trasporti/Matrice-OD2020-Passeggeri/hyqr-mpe2`.

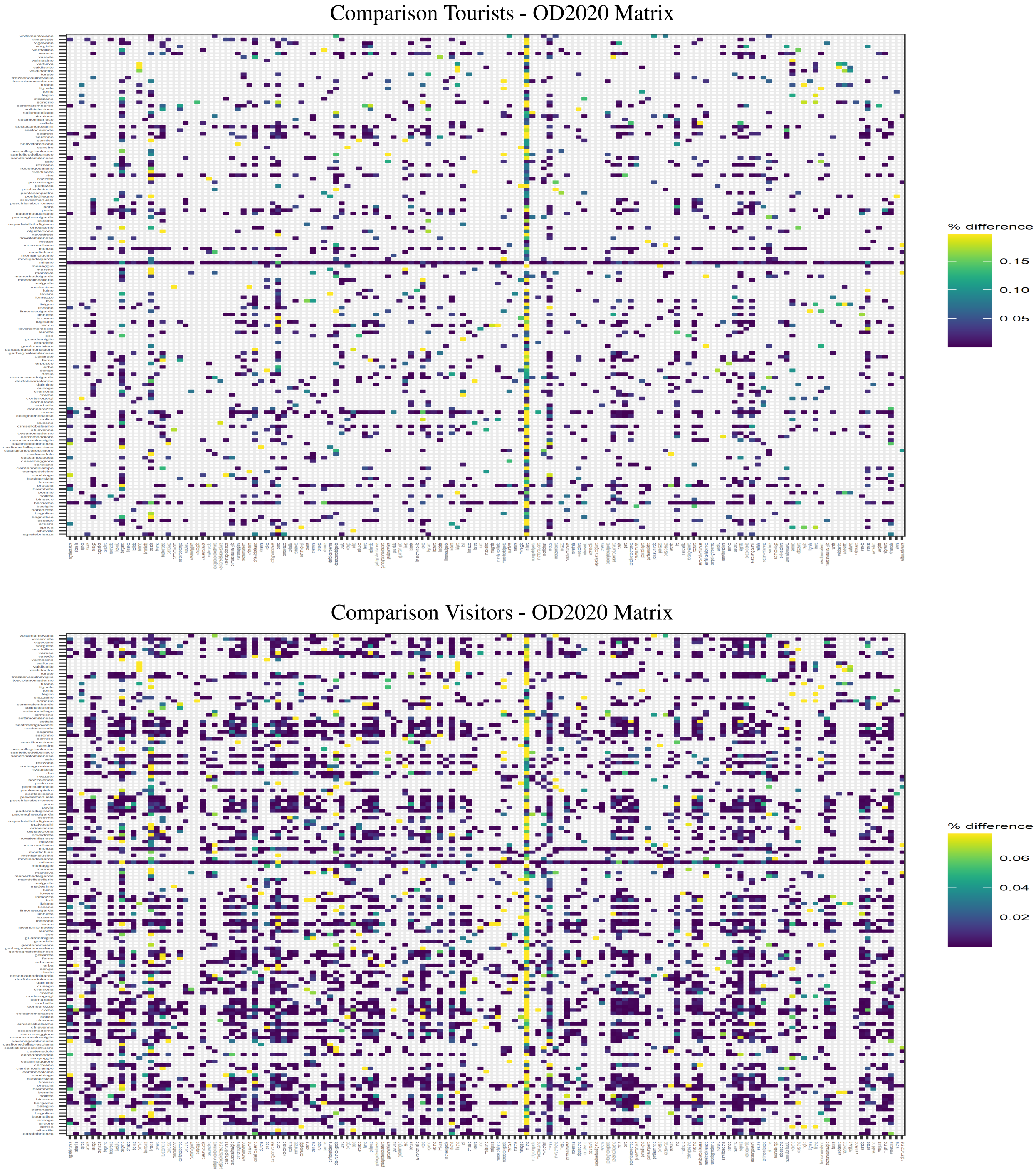

Figure 5. The upper (lower) panel shows the absolute value of the difference between the row standardized adjacency matrix of tourists (visitors) and OD 2020 matrix of commuters. This figure was realized using the R software (4.2.3 version).

2 Centrality Network Indicators

In this section we provide details on how we compute the set of network centrality indicators used as drivers of tourists flows in the gravity model introduced in equation 4 in the Section "Gravity Model". In particular:

- Instrength: it provides information about the flow entering each node j of the network. In particular, given $w_{i,j}$ the flow of tourists from a node i to a node j of the network, this indicator can be defined as:

$$Instrength_j = \sum_i w_{i,j}$$

- Outstrength: it provides information about the flow exiting from each node i of the network. In particular, this indicator can be defined as:

$$Outstrength_j = \sum_j w_{i,j}$$

- Betweenness: it is the number of shortest paths (connecting all the pairs of nodes of the network) that pass through i.

$$Betweenness_i = \frac{\sum_{j,k} n_{j,k}(i)}{n_{j,k}}$$

  where $n_{j,k}(i)$ is the number of shortest paths connecting node j and k passing through node i and $n_{j,k}$ is the number of shortest paths connecting node j and k.

- Authority score: it provides information on the centrality of a node based on the sum of hub score centralities of the neighbours (e.g., a node is important if it is pointed by hub nodes).

$$Authority_i = \alpha_1 * W^t * Hub - score$$

- Hub score: it provides information on the centrality of a node based on the sum of authority score centralities of the neighbours (e.g., a node is important if it points to authority nodes).

$$Hub_i = \alpha_2 * W^t * Authority - score$$

  Therefore, the hub and authority centralities are the eigenvectors of W^tW and WW^t corresponding to same principal eigenvalue $\lambda = \frac{1}{\alpha_1 * \alpha_2}$

- Local Efficiency: it provides information about the relevance of a node i in allowing flow within the network.

$$Nodal\ Efficiency_i = \frac{1}{n-1} \sum_{i,j} \frac{1}{d_{i \neq j}}$$

$$Local\ Efficiency_i = \frac{1}{n} \sum_{i \in G_i} Nodal\ Efficiency(G_i)$$

  where G_i is the subgraph of neighbours of node i.

3 Descriptive statistics drivers of tourists and visitors network centrality variation

Table 2 shows the descriptive statistics of the drivers of centrality variation in tourists and visitors networks.

	Q1	Median	Mean	Q3	Std.dev	Class	Source	Variable Type
Delta Instrength	0.617	0.774	0.602	0.887	0.327	Numerical	Authors calculation	Dependent Variable
Delta Betweenness	-0.607	0.204	-0.941	0.769	0.333	Numerical	Authors calculation	Dependent Variable
Delta efficiency	-0.092	-0.012	-0.027	0.059	0.064	Numerical	Authors calculation	Dependent Variable
Income per taxpayer	17,904	20,518	20,388	22,093	4,356	Numerical	MEF	Independent Variable
Accommodation beds	0.045	0.128	0.731	0.780	0.742	Numerical	Lombardy Open Data	Independent Variable
Cultural heritage	0.000	0.000	0.374	1.000	0.285	Dummy	Lombardy Open Data	Independent Variable
Ski routes	0.000	0.000	0.117	0.000	0.222	Dummy	Lombardy Open Data	Independent Variable
Book shops	0.0001	0.0001	0.0002	0.0003	0.0003	Numerical	Lombardy Open Data	Independent Variable
Methane distributors	0.000	0.000	0.294	1.000	0.257	Dummy	Lombardy Open Data	Independent Variable
Festivals	0.000	0.000	0.313	1.000	0.265	Dummy	Lombardy Open Data	Independent Variable
Farm houses	0.000	1.000	0.595	1.000	0.491	Dummy	Lombardy Open Data	Independent Variable
Intermodal nodes	0.000	0.000	0.037	0.000	0.189	Dummy	Lombardy Open Data	Independent Variable
Natural reserves	0.000	0.000	0.067	0.000	0.152	Dummy	Lombardy Open Data	Independent Variable

Table 2. We highlight the descriptive statistics of the dependent and independent variables used to detect the drivers of centrality variation in tourists and visitors networks.

4 Cluster analysis approach

In this section, we explain the empirical approach we use to cluster Lombardy municipalities in different groups based on a set of social, economic and environmental variables.

In particular, since the local business environment and living conditions are relevant demand side factors influencing the number of tourists flows, we consider the *Income per contribuent*, and the number of *Firms* per inhabitant in the place of origin, consistently with previous studies that use such variables to analyse the main drivers of tourists flows[2–8].

Availability of high quality services may allow to perceive lower fatigue in visiting a specific place[9–14]. We thus plug in our model the number of *Bank offices* per inhabitant.

As environmental risk may reduce the level of perceived safety and significantly affect the number of visitors in specific periods of the year, we account for *Flood risk* (expressed as the portion of population subject to high flood risk) and *Landslide risk* (expressed as the portion of population subject to high landslide risk)[5,6,9]. We also include the percentage of *Waste sorting*, availability of *Drinking water* (expressed as thousands of cubic meters of water per inhabitant dispensed by the local municipality) and portion of *Soil usage* as proxies of urban ecology, quality of local environmental services and of the level of anthropization of the area[7,15].

In terms of demographic variables, we consider *Population density* in line with[4],[5] and[16].

We finally account for potential heterogeneity across municipalities in the social and healthcare sectors. In particular, we include in our analysis the number of *Schools* per inhabitant, the monetary value of expenditures for *Social services* per inhabitant, the number of *Pharmacies* per inhabitant, and number of beds in *Healthcare infrastructures* per inhabitant.

We perform a cluster analysis based on these social, economic, environmental and demographic variables using the "Ward" hierarchical clustering method as a standard approach to perform such analysis[17]. We then assess the stability of our results by comparing the output of alternative clustering algorithms, such as the k-means and other hierarchical clustering methods based on alternative agglomeration methods such as "Single", "Complete", "Average", "Mcquitty", "Median", "Centroid"[18,19].

To select the optimal number of clusters we rely on the silhouette coefficient, allowing to compare intra and inter cluster distances, thus providing insight on the quality of the clustering method output[20]. We define the silhouette as:

$$Silhouette = \frac{1}{N} s_i \tag{1}$$

where s_i is the silhouette of observation i and N is the sample size. In particular, s_i can be computed as:

$$s_i = \frac{b_i - a_i}{max(a_i; b_i)} \tag{2}$$

where a_i is the mean distance of observation i from all other units in the same cluster (c_i) and b_i is the minimum average distance of observation i from all units in other clusters.

In formula:

$$a_i = \frac{1}{N_{c_i} - 1} \sum_{j \in c_i, j \neq i} (d_{i,j}) \tag{3}$$

$$b_i = \min_{c_l \neq c_i} \frac{1}{N_{c_l}} \sum_{l \in c_l} (d_{i,l}) \tag{4}$$

where N_{c_i} is the size of cluster c_i and $d_{i,j}$ is the euclidean distance between observation i and j.

Furthermore, we compare the results of our cluster analysis with the tourism classification of Italian municipalities made by ISTAT in 2020.[3] Based on ISTAT classification our 163 municipalities are allocated to four alternative classes as reported in Figure 6.[4] In particular, they belong to the classes *"Cultural"*, *"Mountain"*, *"Lake"*, *"Not specific"*.

We thus compute the purity index as the percentage of municipalities that are classified by our clustering algorithm in the same tourism class according to the ISTAT analysis. In formula:

[3] Detailed information about the tourism classification of Italian municipalities made by ISTAT in 2020 is available at the following link: `https://www.istat.it/it/files//2020/09/classificazione-turistica-comuni.Istat_.pdf`.

[4] We re-arrange the ISTAT classification, by allocating municipalities with multiple tourism vocation (e.g. "Cultural-Lake") to the prevalent location. Furthermore, Milan, originally classified by ISTAT in the class "Metropolies" is in this case allocated to the "Not specific - (multidimensional)" tourism class, since no other municipality in Lombardy would have been classified in the same group.

$$Purity\ Index = \frac{1}{N}\sum_{k=1}^{K} max_s|c_k \cap t_s| \tag{5}$$

where where N is the number of municipalities, K is the number of clusters, c_k is cluster k and t_s is the classification which has the maximum number of elements in common with cluster c_k.

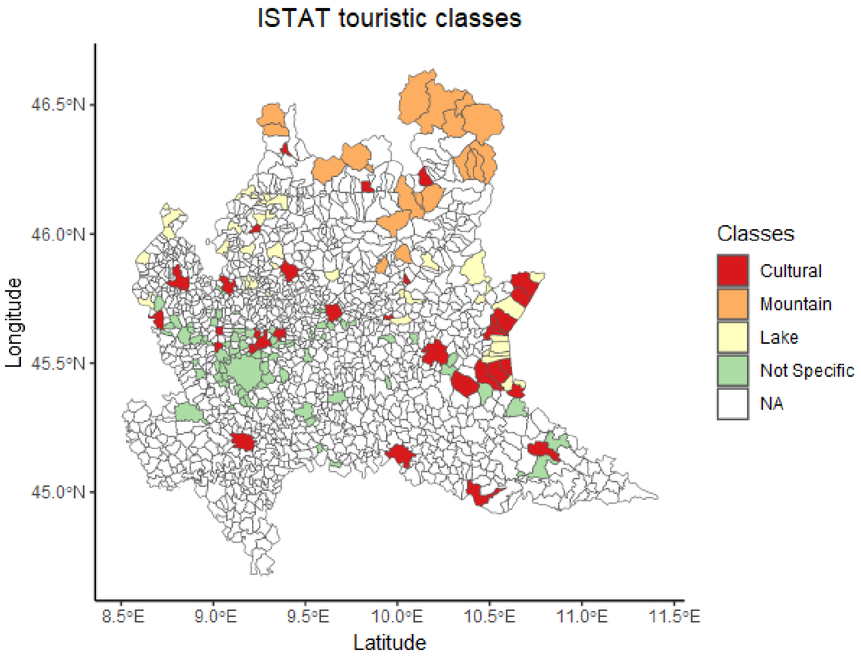

Figure 6. The tourism classification made by ISTAT of Italian municipalities. This figure was realized using the R software (4.2.3 version).

4.1 Cluster analysis results

We first apply the "Ward" agglomeration method[17] and we select results in correspondence of a number of clusters equal to three since it maximizes the value of the silhouette equal to 0.2 (ranging between 0.12 and 0.17 for a number of clusters between 4 and 8. See the upper panel in Figure 7 for further details). Furthermore, for a number of clusters equal to three we also obtain the maximum purity index accounting for 0.7 (ranging between 0.69 and 0.44 for a number of clusters between 4 and 8). Similar results, suggesting three as the optimal number of clusters, are also confirmed by the set of other clustering algorithms including the k-means and other hierarchical clustering methods based on alternative agglomeration approaches such as "Single", "Complete", "Average", "Mcquitty", "Median", "Centroid" (see the lower panel in Figure 7).

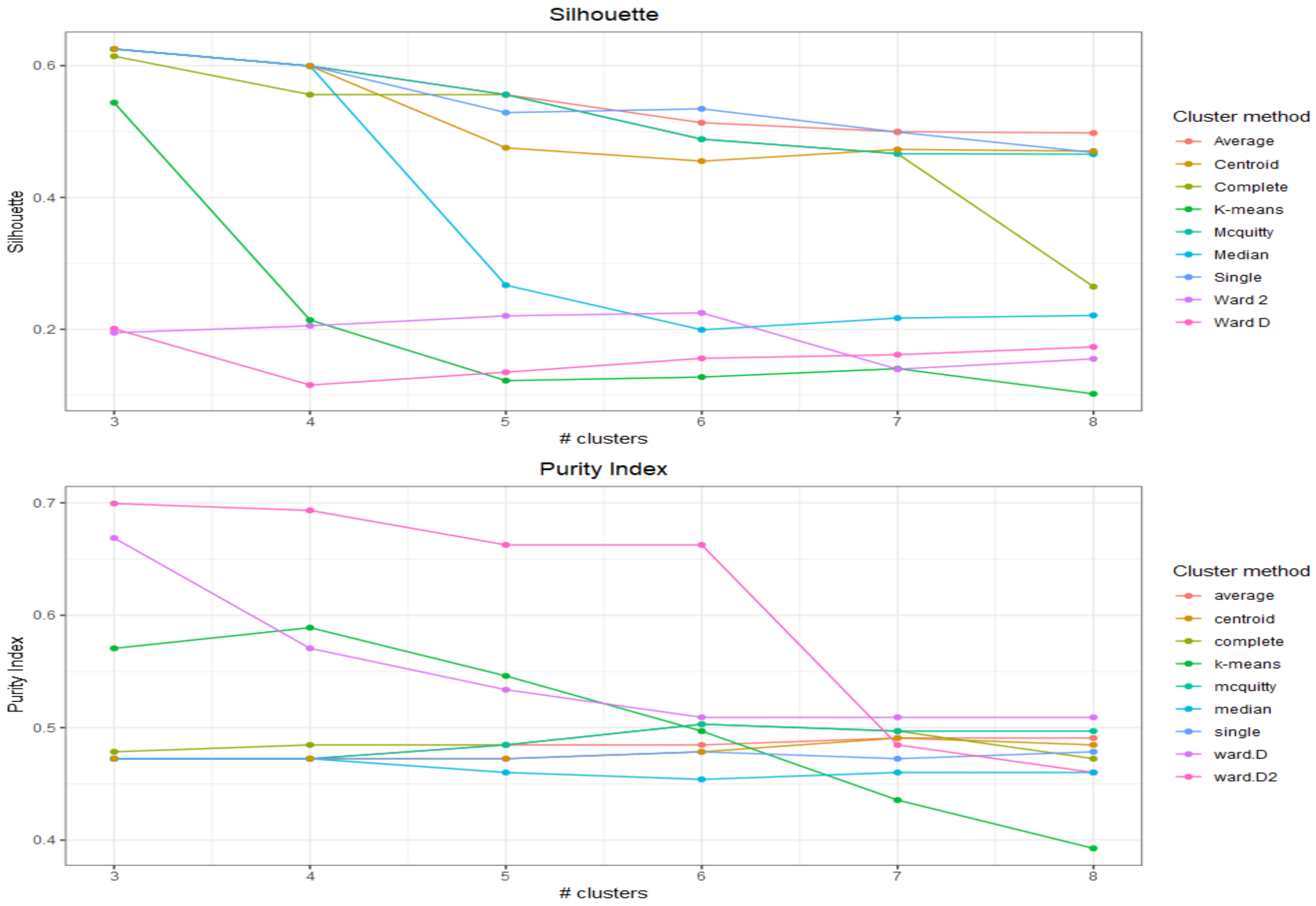

Figure 7. The upper panel shows the optimal number of clusters based on the silhouette criterion across alternative hierarchical and non-hierarchical clustering methods. The lower panel shows the purity index for the same set of hierarchical and non-hierarchical clustering methods for a different number of clusters. We use the four tourism classes identified by ISTAT as the reference classification of municipalities to compute the purity index. This figure was realized using the R software (4.2.3 version).

Figure 8 shows the geographical distribution of Lombardy municipalities across the three identified clusters. Overall, we notice a certain coherence with the four tourism classes identified by ISTAT and reported in Figure 6. Indeed, the first cluster mainly overlaps with the *Cultural* and *Lake* classes, whereas the second and third clusters mainly represent the *Mountain* and the *Not specific* classes, respectively.

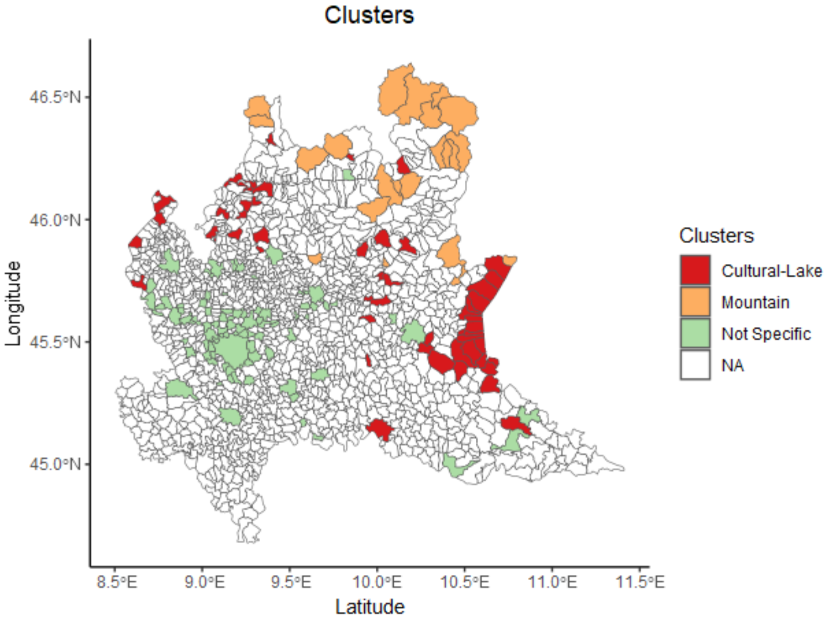

Figure 8. We show the geographical distribution of Lombardy municipality based on the Ward clustering method[17], This figure was realized using the R software (4.2.3 version).

Table 3 shows the average values of the set of social, economic and environmental variables used in the clustering analysis for the three identified groups. We observe that the *Mountain* cluster is characterized by larger landslide and flood risk, while the *Not specific* group accounts for larger income per contribuent, population density, and soil usage. The *Cultural-Lake* cluster exhibits larger social services expenditures and healthcare infrastructures beds.

Table 3. We show the average values of a set of social, economic and environmental variables for the three groups identified through our cluster analysis.

Cluster	$Not-specific$	$Cultural-Lake$	$Mountain$
Income per taxpayer	$22,472$	$18,472$	$16,394$
Soil usage	0.390	0.116	0.026
Waste sorting	0.740	0.706	0.597
Landslide risk	0.001	0.017	0.083
Flood risk	0.010	0.039	0.058
Bank offices	$4.176*10^{-4}$	0.001	0.002
Drinking water	0.105	0.119	0.312
Schools	0.001	0.001	0.002
Pharmacies	$3.163*10^{-4}$	$4.371*10^{-4}$	0.002
Social services	257.282	366.751	116.130
Healthcare infrastructures	$1.353*^{-5}$	$4.318*10^{-5}$	$2.472*10^{-4}$
Population density	$1,944.167$	306.023	65.388
Firms	0.076	0.084	0.168

5 Monthly Gravity Models Results

In this section, Table 4 exhibits the descriptive statistics of the dependent and independent variables used to in the gravity model to analyse if areas receiving high tourists flows are particularly appealing also for visitors, or if instead municipalities tend to attract alternative tourism behaviours.

In addition, Tables 5, 6, 7 and 8 show the coefficients of monthly gravity models.

	Q1	Median	Mean	Q3	Std.Dev	Variable Type
Tourists flow	21	67	67	419	849	Dependent Variable
Income per taxpayer	18,643	21,362	21,362	22,957	4,269	Independent Variable
Population	6,685	16,091	16,091	46,831	51,489	Independent Variable
Instrength	50,087	135,801	135,801	350,995	160,632	Independent Variable
Authority	0.002	0.006	0.006	0.018	0.082	Independent Variable
Oustrength	53,509	127,038	127,038	423,010	254,447	Independent Variable
Hub score	0.002	0.022	0.022	0.102	0.123	Independent Variable
Betweenness	0.004	0.008	0.008	0.013	0.006	Independent Variable
Efficiency	0.027	0.028	0.028	0.028	0.001	Independent Variable
Cultural heritage inside	26.333	355.500	1,144.830	1,859.016	1,600.376	Independent Variable
Ski routes inside	0.000	0.000	185.202	140.669	428.048	Independent Variable
Farm houses inside	50.333	620.000	1,837.961	2,939.726	2,547.994	Independent Variable
Intermodal nodes inside	0.000	58.474	144.253	281.339	179.256	Independent Variable
Methane distributors inside	32.500	489.431	1,143.478	2,050.331	1,385.282	Independent Variable
Festivals inside	29.250	366.583	967.408	1,536.364	1,314.889	Independent Variable
Museums inside	0.000	59.550	176.091	279.591	243.839	Independent Variable
Travel distance	31.000	53.000	69.282	94.757	51.343	Independent Variable

Table 4. We show the descriptive statistics of dependent and independent variables used in the gravity model. We also include a set of covariates describing the availability of touristic services and attractions that are within the travel distance between node i and j. We compute such variables as the product between the average travel distance (from the origin) to reach nodes in between node i and node j and total number of visits to *Museums* in year 2021, *Cultural heritage* items, *Ski routes*, *Farm houses*, *Intermodal nodes*, *Methane distributors* and *Festivals* in the municipalities with a travel distance from the origin lower than that to travel between nodes i and j.

	Dependent variable:					
	Tourists flows					
	(1)	(2)	(3)	(4)	(5)	(6)
Income per taxpayer orig.	−0.045	−0.137	−0.072	−0.054	−0.111	−0.082
	(0.091)	(0.093)	(0.085)	(0.083)	(0.081)	(0.082)
Population orig.	0.459***	0.435***	0.530***	0.560***	0.681***	0.572***
	(0.128)	(0.128)	(0.119)	(0.117)	(0.116)	(0.114)
Instrength orig.	0.002	0.024	−0.005	0.028	0.018	−0.011
	(0.031)	(0.030)	(0.028)	(0.029)	(0.028)	(0.027)
Outstrength orig.	−0.238	−0.327	−0.344	−0.532**	−0.594***	−0.204
	(0.256)	(0.256)	(0.239)	(0.232)	(0.229)	(0.229)
Betweenness orig.	0.024	0.066	0.044	−0.025	0.052	0.003
	(0.050)	(0.051)	(0.047)	(0.046)	(0.045)	(0.045)
Authority orig.	−0.272	−0.416*	−0.178	−0.438**	−0.432**	−0.287
	(0.229)	(0.226)	(0.211)	(0.208)	(0.205)	(0.206)
Hub orig.	1.342***	1.591***	1.785***	1.975***	2.277***	1.578***
	(0.466)	(0.470)	(0.437)	(0.424)	(0.419)	(0.419)
Efficiency orig.	0.031	−0.062	−0.020	−0.026	−0.003	0.001
	(0.055)	(0.054)	(0.050)	(0.049)	(0.049)	(0.050)
Mount clst. orig.	0.147	0.057	−0.028	−0.121	0.095	−0.249*
	(0.155)	(0.160)	(0.153)	(0.151)	(0.147)	(0.147)
Cultural-Lake clst. orig.	0.050	0.151	0.121	0.121	0.151*	−0.074
	(0.098)	(0.103)	(0.093)	(0.094)	(0.091)	(0.092)
Note:						*p<0.1; **p<0.05; ***p<0.01

Table 5. We show the coefficients of drivers of the gravity model. Column 1-6 refers to models estimated for the first six months of the year 2022. Part I.

	Dependent variable:					
	Tourists flows					
	(1)	(2)	(3)	(4)	(5)	(6)
Income per taxpayer dest.	−0.058	−0.033	−0.004	0.100	0.041	0.064
	(0.093)	(0.086)	(0.080)	(0.082)	(0.079)	(0.078)
Population dest.	0.177	0.696***	0.803***	0.766***	0.862***	0.661***
	(0.148)	(0.173)	(0.131)	(0.120)	(0.112)	(0.101)
Instrength dest.	0.038	0.063	−0.016	−0.076	−0.014	0.069**
	(0.081)	(0.075)	(0.065)	(0.087)	(0.048)	(0.030)
Outstrength dest.	−0.088	−1.797***	−1.098**	−0.457	−0.956***	−0.841***
	(0.635)	(0.661)	(0.500)	(0.502)	(0.315)	(0.280)
Betweenness dest.	0.079***	0.031	0.071***	0.074***	0.082***	0.044*
	(0.018)	(0.023)	(0.019)	(0.023)	(0.024)	(0.023)
Authority dest.	−0.189*	−0.527***	−0.467***	−0.319	−0.494***	−0.726***
	(0.110)	(0.163)	(0.167)	(0.235)	(0.163)	(0.151)
Hub dest.	0.746**	1.037***	0.883***	0.900***	1.067***	1.667***
	(0.311)	(0.273)	(0.212)	(0.258)	(0.248)	(0.555)
Efficiency dest.	0.018	0.031*	0.032**	0.026	0.059***	0.004
	(0.017)	(0.018)	(0.016)	(0.017)	(0.017)	(0.020)
Mountain clst. dest	0.995***	1.088***	1.083***	1.000***	0.729***	1.379***
	(0.157)	(0.161)	(0.157)	(0.161)	(0.158)	(0.157)
Cultural-Lake clst. dest.	0.384***	0.490***	0.477***	0.626***	0.534***	0.902***
	(0.105)	(0.115)	(0.103)	(0.112)	(0.093)	(0.097)
Cultural heritage inside	0.054	−0.005	−0.015	−0.031	−0.044	−0.079
	(0.072)	(0.073)	(0.068)	(0.065)	(0.068)	(0.065)
Ski routes inside	0.067	0.046	0.043	−0.004	0.014	0.034
	(0.049)	(0.050)	(0.047)	(0.046)	(0.049)	(0.043)
Farm-houses inside	−0.018	0.003	0.018	0.039	0.016	0.044
	(0.038)	(0.041)	(0.036)	(0.035)	(0.036)	(0.035)
Intermodal nodes inside	−0.031	−0.293	−0.034	−0.012	−0.175	−0.066
	(0.176)	(0.188)	(0.169)	(0.159)	(0.164)	(0.162)
Methane distributors inside	0.008	0.020	0.005	−0.019	−0.008	−0.005
	(0.041)	(0.040)	(0.037)	(0.037)	(0.037)	(0.035)
Festivals inside	−0.097**	−0.059	−0.056	−0.022	−0.025	−0.047
	(0.047)	(0.048)	(0.043)	(0.043)	(0.045)	(0.041)
Museums inside	−0.002	0.005	−0.024	0.045	−0.022	0.033
	(0.106)	(0.107)	(0.099)	(0.096)	(0.098)	(0.097)
Time distance	−0.831***	−0.848***	−1.009***	−0.905***	−0.950***	−0.823***
	(0.074)	(0.077)	(0.069)	(0.069)	(0.070)	(0.067)
Constant	9.643***	7.509***	9.406***	8.327***	10.308***	8.258***
	(1.662)	(1.673)	(1.516)	(1.524)	(1.536)	(1.526)
Observations	1,655	1,663	1,929	1,909	1,949	2,001
R^2	0.340	0.321	0.351	0.366	0.365	0.366
Adjusted R^2	0.329	0.309	0.341	0.357	0.356	0.357
Note:						*p<0.1; **p<0.05; ***p<0.01

Table 6. We show the coefficients of drivers of the gravity model. Column 1-6 refers to models estimated for the first six months of the year 2022. Part II.

	Dependent variable:					
	Tourists flows					
	(7)	(8)	(9)	(10)	(11)	(12)
Income per taxpayer orig.	−0.120	−0.073	0.059	−0.019	−0.111	0.041
	(0.083)	(0.091)	(0.081)	(0.080)	(0.082)	(0.082)
Population orig.	0.661***	0.435***	0.693***	0.625***	0.645***	0.651***
	(0.113)	(0.121)	(0.115)	(0.115)	(0.121)	(0.119)
Instrength orig.	0.008	0.009	−0.030	0.025	−0.023	0.016
	(0.027)	(0.029)	(0.027)	(0.028)	(0.029)	(0.028)
Outstrength orig.	−0.495**	−0.067	−0.297	−0.517**	−0.409*	−0.633***
	(0.229)	(0.240)	(0.229)	(0.233)	(0.241)	(0.239)
Betweenness orig.	0.016	−0.024	−0.011	0.030	0.052	0.003
	(0.044)	(0.046)	(0.045)	(0.044)	(0.046)	(0.045)
Authority orig.	−0.373*	−0.485**	−0.189	−0.506**	−0.083	−0.359*
	(0.202)	(0.216)	(0.204)	(0.203)	(0.213)	(0.209)
Hub orig.	2.079***	1.464***	2.015***	2.085***	2.072***	2.117***
	(0.415)	(0.444)	(0.423)	(0.416)	(0.440)	(0.432)
Efficiency orig.	0.021	−0.013	0.062	0.024	−0.016	0.082
	(0.050)	(0.052)	(0.048)	(0.048)	(0.050)	(0.060)
Mountain clst. orig.	−0.382**	−0.348**	0.022	0.078	−0.019	−0.188
	(0.154)	(0.163)	(0.150)	(0.155)	(0.156)	(0.154)
Cultural-Lake clst orig.	−0.130	−0.008	0.049	0.087	0.128	0.007
	(0.089)	(0.094)	(0.091)	(0.090)	(0.094)	(0.093)
Note:						*p<0.1; **p<0.05; ***p<0.01

Table 7. We show the coefficients of drivers of the gravity model. Column 7-12 refers to models estimated for six months in the second half of the year 2022. Part I.

	Dependent variable:					
	Tourists flows					
	(7)	(8)	(9)	(10)	(11)	(12)
Income per taxpayer dest.	−0.015	−0.255***	−0.017	0.106	0.141*	0.106
	(0.080)	(0.083)	(0.080)	(0.079)	(0.084)	(0.082)
Population dest.	0.549***	0.276***	0.517***	0.568***	0.292**	0.338***
	(0.092)	(0.076)	(0.093)	(0.108)	(0.130)	(0.129)
Instrength dest.	0.114***	0.030	0.093***	0.108**	0.090**	−0.053
	(0.039)	(0.036)	(0.034)	(0.051)	(0.040)	(0.071)
Outstrength dest.	−0.822***	0.239	−1.035***	−1.900***	−0.627	−0.577
	(0.285)	(0.253)	(0.256)	(0.369)	(0.394)	(0.603)
Betweenness dest.	0.024	0.133***	0.049***	0.029	0.078***	0.060***
	(0.025)	(0.025)	(0.018)	(0.020)	(0.020)	(0.016)
Authority dest.	−0.807***	−0.325***	−0.634***	−0.315**	−0.405**	0.073
	(0.150)	(0.119)	(0.189)	(0.159)	(0.204)	(0.093)
Hub dest.	0.643***	−0.161	2.242***	1.498***	0.888*	1.214*
	(0.185)	(0.243)	(0.562)	(0.260)	(0.477)	(0.684)
Efficiency dest.	0.095***	0.058***	0.065***	0.122***	0.064***	0.070***
	(0.020)	(0.022)	(0.022)	(0.017)	(0.016)	(0.017)
Mountain clst. dest.	1.203***	1.084***	0.732***	0.454***	0.493***	1.124***
	(0.142)	(0.148)	(0.158)	(0.158)	(0.170)	(0.143)
Cultural-Lake clst. dest.	0.668***	0.701***	0.498***	0.385***	0.289***	0.305***
	(0.100)	(0.101)	(0.095)	(0.097)	(0.105)	(0.106)
Cultural heritage inside	−0.048	−0.018	0.017	−0.105	0.090	0.121*
	(0.063)	(0.068)	(0.067)	(0.066)	(0.074)	(0.066)
Ski routes inside	−0.081*	0.042	0.015	0.023	0.067	0.115**
	(0.043)	(0.044)	(0.047)	(0.045)	(0.051)	(0.045)
Farm-houses inside	0.061*	0.029	0.029	0.037	−0.007	−0.022
	(0.035)	(0.035)	(0.036)	(0.036)	(0.039)	(0.036)
Terminal nodes inside	−0.032	−0.046	−0.293*	−0.404**	−0.328*	−0.073
	(0.162)	(0.171)	(0.161)	(0.167)	(0.176)	(0.160)
Methane distributors inside	−0.053	0.003	0.041	0.049	0.091**	0.086**
	(0.035)	(0.038)	(0.037)	(0.037)	(0.040)	(0.037)
Festivals inside	0.016	−0.084*	−0.060	−0.013	−0.105**	−0.098**
	(0.042)	(0.045)	(0.043)	(0.042)	(0.048)	(0.042)
Museums inside	-0.051	0.151	−0.001	−0.010	−0.091	−0.024
	(0.092)	(0.095)	(0.092)	(0.096)	(0.103)	(0.097)
Time distance inside	−0.864***	−0.757***	−0.890***	−0.967***	−0.864***	−0.875***
	(0.067)	(0.070)	(0.068)	(0.069)	(0.073)	(0.073)
Constant	11.601***	9.319***	11.689***	12.330***	9.299***	12.202***
	(1.504)	(1.584)	(1.532)	(1.506)	(1.538)	(1.519)
Observations	1,986	1,743	1,972	1,967	1,894	1,985
R^2	0.377	0.349	0.359	0.373	0.341	0.334
Adjusted R^2	0.368	0.338	0.350	0.364	0.331	0.324
Note:						*p<0.1; **p<0.05; ***p<0.01

Table 8. We show the coefficients of drivers of the gravity model. Column 7-12 refers to models estimated for six months in the second half of the year 2022. Part II.

References

1. Nyns, S. & Schmitz, S. Using mobile data to evaluate unobserved tourist overnight stays. *Tourism Management* **89**, 104453 (2022).
2. Prideaux, B. Factors affecting bilateral tourism flows. *Annals Tourism Research* **32**, 780–801 (2005).
3. Zhang, J. & Jensen, C. Comparative advantage: explaining tourism flows. *Annals tourism research* **34**, 223–243 (2007).
4. Lorenzini, E., Calzati, V. & Giudici, P. Territorial brands for tourism development: A statistical analysis on the marche region. *Annals Tourism Research* **38**, 540–560 (2011).
5. Massidda, C. & Etzo, I. The determinants of italian domestic tourism: A panel data analysis. *Tourism Management* **33**, 603–610 (2012).
6. Giambona, F. & Grassini, L. Tourism attractiveness in italy: Regional empirical evidence using a pairwise comparisons modelling approach. *International Journal Tourism Research* **22**, 26–41 (2020).
7. Ma, X., Yang, Z. & Zheng, J. Analysis of spatial patterns and driving factors of provincial tourism demand in china. *Scientific Reports* **12**, 2260 (2022).
8. Xu, D., Zhang, J.-H., Huang, Z., Zhou, Y. & Fan, Q. Tourism community detection: A space of flows perspective. *Tourism Management* **93**, 104577 (2022).
9. Cracolici, M. F. & Nijkamp, P. The attractiveness and competitiveness of tourist destinations: A study of southern italian regions. *Tourism management* **30**, 336–344 (2009).
10. Liu, Y., Shi, J. & Jian, M. Understanding visitors' responses to intelligent transportation system in a tourist city with a mixed ranked logit model. *Journal Advanced Transportation* **2017** (2017).
11. Lewis, C. & D'Alessandro, S. Understanding why: Push-factors that drive rural tourism amongst senior travellers. *Tourism Management Perspectives* **32**, 100574 (2019).
12. Sun, J. *et al.* Development and validation of a tourism fatigue scale. *Tourism Management* **81**, 104121 (2020).
13. Kim, E.-J., Kim, Y., Jang, S. & Kim, D.-K. Tourists' preference on the combination of travel modes under mobility-as-a-service environment. *Transportation Research Part A: Policy Practice* **150**, 236–255 (2021).
14. Simini, F., Barlacchi, G., Luca, M. & Pappalardo, L. A deep gravity model for mobility flows generation. *Nature communications* **12**, 6576 (2021).
15. Song, C., Qu, Z., Blumm, N. & Barabási, A.-L. Limits of predictability in human mobility. *Science* **327**, 1018–1021 (2010).
16. Pompili, T., Pisati, M. & Lorenzini, E. Determinants of international tourist choices in italian provinces: A joint demand–supply approach with spatial effects. *Papers Regional Science* **98**, 2251–2273 (2019).
17. Murtagh, F. & Legendre, P. Ward's hierarchical agglomerative clustering method: which algorithms implement ward's criterion? *Journal classification* **31**, 274–295 (2014).
18. McQuitty, L. L. Similarity analysis by reciprocal pairs for discrete and continuous data. *Educational Psychological measurement* **26**, 825–831 (1966).
19. Hartigan, J. A. Clustering algorithms, new york: John willey and sons. *Inc. Pages113129* (1975).
20. Rousseeuw, P. J. Silhouettes: a graphical aid to the interpretation and validation of cluster analysis. *Journal computational applied mathematics* **20**, 53–65 (1987).
